# Validity evidence of the Brazilian version of the Assessment Interprofessional Collaborative Leadership Scale*

**DOI:** 10.1590/1518-8345.7894.4719

**Published:** 2025-11-17

**Authors:** Natalia Dalforno da Silva, Geisa Colebrusco de Souza Gonçalves, Jaqueline Alcantara Marcelino da Silva, Flávio Rebustini, Alexandre Pazetto Balsanelli

**Affiliations:** 1 Universidade Federal de São Paulo, Escola Paulista de Enfermagem, São Paulo, SP, Brazil.; 2 Hospital Sírio Libanês, São Paulo, SP, Brazil.; 3 Universidade Federal de São Carlos, Departamento de Enfermagem, São Carlos, SP, Brazil.; 4 Universidade de São Paulo, Escola de Ciências, Humanidades e Artes, São Paulo, SP, Brazil.

**Keywords:** Leadership, Patient Care Team, Interprofessional Relations, Validation Study, Health Personnel, Healthcare Crew Resource Management

## Abstract

to seek evidence of validity of the Assessment Interprofessional Collaborative Leadership Scale for Brazilians, in Portuguese, and to analyze evidence of content, response process and internal structure.

psychometric evaluation study, with translation, back-translation, and evaluation by a committee of experts regarding linguistic and content equivalence. The pre-test was carried out with 40 participants and, subsequently, the final version was applied to 318 health workers. Exploratory factor analysis and reliability testing were performed using Cronbach’s alpha and McDonald’s omega.

in the content validation, four items presented scores below the critical value. Of the participants, the majority were nurses (81%) and physiotherapists (10%), and were female (79%). The factorability obtained was Kaiser-Meyer-Olkin = 0.94 and Bartlett’s sphericity = 2991.4 with p < 0.05. Parallel analysis indicated a one-dimensional model, with variance explained by 63.71%. The model’s adequacy indices showed satisfactory levels. And adequate reliability indices (Cronbach’s alpha = 0.97 and McDonald’s omega = 0.97).

the Interprofessional Collaborative Leadership Assessment Scale, with a unidimensional structure and 28 items, presented good evidence of content validity, response process and internal structure, considered adequate to measure interprofessional collaborative leadership.

## Highlights

**(1)** Instrument to measure interprofessional collaborative leadership. **(2)** Instrument to implement an interprofessional care model. **(3)** Diagnosis to support interprofessional development programs. **(4)** Evidence of validity of an interprofessional collaborative leadership instrument.

## Introduction

Collaborative interprofessional practice is a form of work organization that is essential for addressing complex health issues and providing comprehensive care^(1)^. It requires coordinated efforts to ensure the sharing of information and decisions so that the expertise of each team member is made viable in the implementation of care and in meeting health needs^(2)^.

Among the competencies of collaborative interprofessional practice is collaborative leadership, in which professionals work together with patients and families to promote shared decision-making and produce co-responsibilities within the team^(2)^.

Unlike traditional, hierarchical and centralized leadership, interprofessional collaborative leadership is established in the sharing of responsibilities, collective and articulated construction of solutions from multiple perspectives, of professionals and patients, in clinical decisions to improve the quality of care^(3)^. Also described in the literature as collective or distributive leadership, it is considered a strategy to implement complex interventions, promote safe communication and participation, and involve stakeholders, in an environment in which equity is sought^(4)^. However, its practical application in the daily routine of services remains little explored.

In this scenario, the Assessment Interprofessional Collaborative Leadership Scale (AICLS)^(5)^ instrument was developed and published in the Canadian context in 2019, with the proposal of measuring interprofessional collaborative leadership in health institutions, through the individual perceptions of the health professionals who make up the teams^(5)^. The genesis of its construction is the theoretical model that explores the interrelationship between vertical and formal structures in the institution and collaborative teams, as new possibilities for the exercise of leadership^(6)^.

Although the contexts of training and practice in health in Brazil and Canada present local specificities, the literature on interprofessional teamwork has broadly established the interprofessional competencies necessary for these to occur, such as communication, collaboration and collaborative leadership, in various health settings^(7)^. Specific studies on interprofessional collaborative leadership are scarce, and a gap has been identified in the measurement instruments. The Clinical Shared Leadership Scale (CSLS)^(8)^ is recognized; however, it does not assess shared decision-making with direct involvement of patients and caregivers, a fundamental characteristic identified in this competency, as found in research with the significant participation of young people in the proposal and development of integrated care, based on interprofessional collaborative leadership^(9)^. Thus, interprofessional collaborative leadership requires the active participation of patients and family (service users), which is why the translation of the AICLS^(5)^ was chosen, given its advancement and theoretical relevance to the reality and context of the Brazilian health system.

The AICLS instrument, in Canada, underwent content validity analysis and was considered highly relevant in its preliminary version, its reliability evidence was 0.96, and its dimensions ranged from 0.85 to 0.92 [Symbiotic relationship = 0.87; Mindfulness = 0.92; Shared resources = 0.92 and Capacity to lead = 0.85]^(5)^.

Therefore, it is important to translate and validate this instrument, with the premise that it is an effective measure for analyzing collaborative leadership in interprofessional teams and that it can impact the understanding of this competence to achieve improvements in the quality and safety of care offered by the teams. In this sense, the objective of this study was to seek evidence of validity of the Assessment Interprofessional Collaborative Leadership Scale (AICLS) for Brazilian Portuguese and to analyze evidence of content, response process and internal structure.

## Method

### Study design

This is a psychometric evaluation study, of validity evidence and cross-cultural adaptation (CCA)^(10)^ through the following phases: translation; synthesis, back-translation, evaluation by a committee of experts, pre-testing of the instrument and, finally, analysis of the evidence of validity of the instrument^(10-11)^. The study followed the guidelines set out in the Test Adaptation Reporting Standards (TARES)^(12)^.

The translation was carried out by two independent translators, with a single synthesis version prepared by a third translator^(10)^, evaluated and approved by the author of the instrument.

The equivalence of the Portuguese version was established based on four dimensions: semantic, idiomatic, cultural and conceptual by 17 experts in collaborative interprofessional practice and/or translations of measurement instruments. After approval by this committee of experts, the pilot test was carried out with 40 care or management workers, who made up teams in hospital inpatient units.

To assess the psychometric properties, the use and adoption of the AICLS^(5)^ instrument in Brazil, we sought to gather evidence of validity based on content, response process, internal structure and reliability verification.

Content validation consists of a qualitative assessment (expert committee) and a quantitative assessment of each item, considering the individual importance of the item in the composition of the instrument and representation of the construct^(13)^, by calculating the Content Validity Ratio (CVR)^(14)^.

In the validation of the response process, we verify how the item is interpreted and its relationship with the measured construct^(11)^. Participants in the pilot test analyzed the viability, comprehensibility and ease of use of the instrument. Response time was recorded, as this data may provide evidence of the complexity of the response processes^(11,15-16)^.

The analysis of the internal structure of the construct was performed by Exploratory Factor Analysis (EFA), which assesses, through factor loadings, the relevance of each item to the factor^(16)^. Factor retention substantially influences EFA results, and also has strong theoretical implications, as it predetermines the assumed dimensionality of a latent construct. Due to its exploratory nature, EFA is used in cases where understanding of the factorial structure of the latent concept is still incipient^(17)^, as is the case of AICLS, recently described in the literature^(5)^. Adjustment indices of the factorial solution were used to support the determination of the number of retained factors^(17)^, and the adequacy of the factorial structure found^(18)^.

Reliability was also verified, using two indicators, as recommended in the literature, to increase the reliability of the interpretation^(19)^.

### Setting

The study was developed in a large, private, tertiary-level general hospital located in the city of São Paulo (SP), Brazil, considered an international reference center in health.

### Study population and sample

The study population consisted of healthcare workers in management and care positions who were part of interprofessional teams in critical care units and inpatient, clinical and surgical units. The sample size was calculated based on the recommendations for verifying the internal structure of instruments, which indicate a minimum number of 300 participants^(13)^. In this study, a convenience sample of 318 healthcare workers was obtained, comprising nurses, nursing technicians, physiotherapists, doctors, pharmacists, psychologists and nutritionists. The inclusion criterion was workers who had worked at the institution for at least one year, in management or care positions, and shared activities with at least one worker from another area of training. The exclusion criterion used was workers who were on leave at the time of data collection.

### Study variables

The AICLS includes 28 items, divided into four dimensions: Symbiotic relationship; Mindfulness; Shared resources; and Ability to lead^(5)^.

The Symbiotic relationship dimension, composed of five items, is defined by a collaboration in which all team members have their roles well established and adapt reciprocally to the demands that arise^(5)^.

The Mindfulness dimension is composed of nine items, characterized by attentive and extended focus, with attention to immediate situations and experiences as they arise^(5)^.

The Shared resources dimension is composed of seven items, defined by an environment that encourages openness among professionals to share knowledge, skills, and expertise within a team^(5)^.

The Ability to lead dimension, composed of seven items, is defined by a willingness to lead and accept responsibility for the leadership role^(5)^.

Respondents indicate their general level of agreement with the occurrence of each item on a five-point Likert-type rating scale: 1 - Never; 2 - Rarely; 3 - Occasionally; 4 - Most of the time; and, 5 - Always. The ratings produce scores from 28 to 140^(5)^.

### Data collection

The version produced after the content validation and response process stages was transformed into an electronic questionnaire, along with questions to characterize the sample. Data were collected through the Research Electronic Data Capture (RedCap) platform^(20)^. The invitation to participate was sent via email to the area coordinators, who forwarded it to the health professionals/workers in their respective teams.

### Duration

The data collection period took place between July 15th and September 15^th^, 2023.

### Data processing and analysis

To start the EFA and verify the factorability of the data, the Kaiser-Meyer-Olkin (KMO) index was calculated and the Bartlett Sphericity Test (TEB) was performed^(13)^.

To extract factors, the Unweighted Least Squares (ULS) was used, with oblimin rotation, from a polychoric correlation matrix^(13)^. Subsequently, the dimensionality of the instrument was explored, through the parallel analysis technique, Optimal Implementation of Parallel Analysis (PA), an approach recommended for a set of ordered variables with polytomous scoring^(21)^, Hull method with Comparative Fit Index^(22)^ and application of Closeness of dimensionality^(23)^, composed of three indicators: Item Unidimensional Congruence (I-UniCo), Item Explained Common Variance (I-ECV) and Mean of Item Residual Absolute Loadings (MIREAL). Values of I-UNICO > 0.95, I-ECV > 0.85 and MIREAL < 0.30 suggest that the data can be treated as essentially one-dimensional^(23)^.

The factor solution was applied via structural equation modeling and to verify the need for adjustments the following indices were calculated: Root Mean Square Error of Approximation (RMSEA); Non-Normed Fit Index (NNFI); CFI (Comparative Fit Index); Goodness of Fit Index (GFI); Adjusted Goodness of Fit Index without diagonal values (AGFI)^(24)^. Reliability was verified using Cronbach’s alpha and McDonald’s omega indices^(19)^.

All analyses were performed using the Factor software.

### Ethical aspects

The ethical aspects of the research were followed according to Resolution no. 466 of 2012^(25)^, both in the evaluation phase of the expert committee and in the application of the pilot test and pre-test. These steps took place after approval by the Research Ethics Committee (CEP) of the Federal University of São Paulo and the co-participating institution, the hospital where the research was conducted, under Opinions no. 5,533,152 and no. 5,571,337, respectively.

## Results

### Cross-cultural adaptation and content validation

The equivalence analysis resulted in 62.5% of the 28 items having an agreement rate greater than 80%. After the first round of analysis by the expert committee, 12 items that had an agreement rate lower than 0.80 were revised, besides that, the name of the instrument was also altered, the first two explanatory sentences of the introduction and the final paragraph.

The suggestions were accepted and, after the modifications, a new round was held with the expert committee to review these items and choose, between two options, the best alternative, taking into account its concepts and meanings within Brazilian culture. The final definition of the 12 items is described in Figure 1.

**Figure 1-:** Modifications made by the authors to the items after suggestions and the second round of the expert committee. São Paulo, SP, Brazil, 2022

**Item after modification**	**Modification**
Section 1: SYMBIOTIC RELATIONSHIP: Symbiotic relationship is a collaboration in which all team members have their own well-established roles and mutually adapt to the dynamics of changing demands.	Changed the word “ *ambos* ” to “ *todos* ” and changed the position of the word “ *dinâmica* ”
2. Encourages team members to value each other’s expertise.	The word “ *competências* ” was replaced by “expertise”
3. Encourages team members to bring their complementary capabilities (sharing knowledge, skills, and experiences) together to direct the care plan.	The word “ *explorar* ” was changed to “ *aproximar* ”, the order of the words “ *compartilhando conhecimentos* ” was reversed, and “ *abordar o planejamento* ” was changed to “ *direcionar o plano* ”.
Section 2: MINDFULNESS: Mindfulness is the sustained, conscious focus of attention on immediate experiences as they happen.	Added “Mindfulness” and changed the word “ *intencional* ” to “ *consciente* ”
7. Encourages team members to focus beyond the status quo (i.e., the usual way of doing things) on relevant and essential issues of care.	Changed “ *ess* ê *ncia* ” to “ *essenciais* ”
8. Encourages members to consider creative solutions for planning complex patient/client care.	Replaced “ *atendimentos mais* ” with “ *cuidados* ”
11. Is receptive to supporting changes suggested by team members.	The order of the word “ *apoio* ” has been inverted.
16. Encourages team members to set shared goals for teamwork.	Changed “ *sobre seu* ” to “ *para o* ”
21. Shares work among team members, according to their capabilities, when care plans are implemented.	Inverted word order at the beginning of the sentence.
22. Team members support patients/clients as collaborative leaders.	Changed “ *apoiem* ” to “ *apoiam* ”
24. All team members accept and take responsibility for their shared teamwork	Replaced “ *responsabilidade* ” with “ *se responsabilizam* ”
27. There is support for rotating the team leader according to the needs of our care planning.	Changed “ *apoiemos* ” to “ *há apoio* ” and “ *conforme* ” to “ *de acordo* ”.

In the quantitative content validation, the critical value of the CVR acceptable to 17 experts was 0.529^(26)^ and indicated the item’s adherence in this study. Four items obtained scores below 0.529 in some of the evaluation categories: theoretically relevant, practically pertinent, sufficiently comprehensive and understandable in the set and dimensionality.

Item 2, in section 1, “Encourages team members to focus beyond the status quo (i.e., the usual way of doing things) on relevant and essential issues of care”, presented a score below 0.529 when its theoretical relevance, practical relevance and scope were evaluated.

Item 19, in section 3, “The decision-making process focuses on shared goals of all team members”, presented a lower score in the scope evaluation.

In section 4, two items obtained lower scores than 0.529. Item 22 “Team members support patients/clients as collaborative leaders” was rated with lower scores in practical relevance, comprehensiveness and dimensionality, and item 23 “Team members are willing to take on team leadership roles when asked” in comprehensiveness and dimensionality. All items were kept in the instrument at this time, since they presented values below the ratio considered in some of the categories evaluated and not in their entirety.

### Validation of the response process

The validity of the response process, carried out through interviews in the pilot test, confirmed the viability of the instrument for use. Participants spent an average of nine minutes and 27 seconds responding to the instrument. The standard deviation was three minutes and fifteen seconds, and the maximum response time was 15 minutes and 51 seconds.

Respondents had difficulty with the term “status quo” in item 7, in section 2, despite the explanation being in parentheses within the item itself, as indicated by the expert committee. As a suggestion, the term “status quo” was removed, and this item was defined as “Encourages team members to develop processes that lead to the creation of an environment where decision-making is shared”.

The understanding of the response options for the items was adequate for 100% of participants, as was the general organization of the items. In the assessment of the scope of the items, 87.5% of participants considered it adequate, and one of them suggested removing items that he considered similar.

Regarding the difficulty in completing the instrument, 62.5% of the participants reported no difficulties and 20 suggestions were reported and considered. Thus, eight items were adjusted (items 1 and 3 in section 1; items 7, 9 and 10 in section 2; items 19 and 21 in section 3; and item 25 in section 4).

The final version of the instrument after the suggested changes was answered by 318 participants, of which six indicated the option of having been at the institution for less than a year, which would have constituted their exclusion, after data collection. However, due to the small percentage (1.8%) in relation to the total sample, it was decided to maintain this in the presentation of the results.

As for the sociodemographic characteristics, it is noteworthy that the sample was mostly composed of females (79%) and in the age range of 31-40 years (44%). A weekly workload of 30-40 hours was prevalent (67%). Regarding the highest qualification, 53% of participants had a specialization. Nurses (44%), followed by nursing technicians (37%) and physiotherapists (10%), were the professionals with the highest participation. Regarding the time of professional experience, the range of 11 to 15 years was predominant (24%) and the time of experience in the unit was one to three (31% of participants). Regarding the type of unit where they worked, the participants worked in the Semi-Intensive Unit (26%), followed by the Intensive Care Unit (21%).

### Validation of the internal structure

The EFA was performed based on the polychoric matrix and it was verified whether the items were factorizable using the Measure of Sample Adequacy (MSA). In the initial modeling using the 28 items of the instrument, good adequacy indices were found, being TEB: 2991.4 (gl = 378; P = 0.000010) and KMO: 0.94977 with bootstrap 95% Confidence Interval (CI) of KMO = (0.573 - 1.204).

The first analysis in the study of the dimensionality/factors of the instrument was performed by parallel analysis (PA), which indicated the existence of only one dimension, with explained variance of 63.71% of the latent variable. Due to the divergence between the original model and that obtained by the PA, additional dimensionality tests were chosen.

The second technique used was the Hull with the CFI, shown in Table 1.

Again, the result indicated only one dimension. The third test adopted was the unidimensionality/multidimensionality technique, with the UniCo, ECV and REAL indexes shown below in Table 2.

**Table 1-:** Dimensionality by Hull with Comparative Fit Index. São Paulo, SP, Brazil, 2023

**Factor numbers**	**CFI** *	**Degrees of freedom**	**Scree test**
0	0.00	378	0.00
1	0.99	350	**250.61** *
2	1.00	323	0.00
3	1.00	297	

*CFI = Comparative Fit Index

**Table 2-:** Closeness of dimensionality. São Paulo, SP, Brazil, 2023

**Variables**		**I-UniCo** *	**I-ECV** ^†^	**I-REAL** ^‡^
V ^§^ 1. Helps members value their contributions to teamwork.		0.99	0.93	0.22
V ^§^ 2. Encourages team members to value each other’s expertise.		0.99	0.92	0.23
V ^§^ 3. Encourages team members to bring together their complementary capabilities (shared knowledge, skills, and experiences) to direct the care plan.		1.00	0.97	0.14
V ^§^ 4. Allows all team members an opportunity to express their opinions.		1.00	0.98	0.11
V ^§^ 5. Enables team members to see their shared results as meaningful and valuable.		0.99	0.95	0.20
V ^§^ 6. Encourages team members to develop processes that lead to the creation of an environment where decision-making is shared.		1.00	0.99	0.08
V ^§^ 7. Encourages team members to focus beyond the usual way of doing things, on relevant and essential issues of care.		1.00	0.98	0.13
V ^§^ 8. Encourages members to consider creative solutions for planning complex patient/client care.		0.99	0.97	0.16
V ^§^ 9. Encourages team members to reevaluate traditional ways of dealing with similar situations.		1.00	0.98	0.11
V ^§^ 10. Encourages open discussions among team members about care planning issues.		1.00	0.99	0.04
V ^§^ 11. Is receptive to supporting changes suggested by team members.		0.99	0.97	0.15
V ^§^ 12. Encourages team members to adapt to different situations.		1.00	0.98	0.11
V ^§^ 13. Encourages team members to question anything that doesn’t make sense.		1.00	0.97	0.12
V ^§^ 14. Supports creative innovation of team members in situations where there is uncertainty in patient/client care planning.		1.00	0.98	0.10
V ^§^ 15. Ensures that all team members have the opportunity to share their perspectives on patient/client care planning.		1.00	0.98	0.13
V ^§^ 16. Encourages team members to set shared goals for teamwork.		1.00	0.99	0.05
V ^§^ 17. Facilitates adjustments of team member roles to situational needs.		1.00	0.99	0.03
V ^§^ 18. Encourages team members to take responsibility for their contributions to the team’s decision-making process.		1.00	0.99	0.06
V ^§^ 19. Focuses on shared goals of all team members in the decision-making process.		1.00	0.97	0.14
V ^§^ 20. Encourages the integration of perspectives to facilitate shared decision-making processes in developing the patient/client care plan.		1.00	0.99	0.06
V ^§^ 21. Shares work among team members, according to their capabilities, when care plans are implemented.		1.00	0.99	0.05
V ^§^ 22. Team members support patients/clients as collaborative leaders.		0.98	0.83	0.26
V ^§^ 23. Team members are willing to take on a leadership role when asked.		0.93	0.72	0.43
V ^§^ 24. All team members accept and take responsibility for their shared teamwork.		0.86	0.62	0.54
V ^§^ 25. All team members accept and take responsibility for their shared teamwork.		0.76	0.54	0.58
V ^§^ 26. Team members mentor each other so that everyone is able to lead the team effectively.		0.94	0.74	0.43
V ^§^ 27. There is support for rotation of the team leader, according to the needs of our care planning.		0.98	0.85	0.30
V ^§^ 28. We select our team leader together.		0.99	0.88	0.23
Assessment	General			
UniCo ^||^	=	0.98		
ECV ^¶^	=	0.92		
MIREAL**	=	0.19		

*I-UniCo = Item One-Dimensional Congruence; ^†^I-ECV - Item Explained Common Variance; ^‡^I-REAL = Item Residual Absolute Loadings; ^§^V = Variable; ^||^UniCo = Unidimensional Congruence; ^¶^ECV = Explained Common Variance; **MIREAL = Mean of Item Residual Absolute Loadings

Based on the results presented, the general model can once again be considered unidimensional, with index values for items 23, 24, 25 and 26 not supporting unidimensionality (REAL-I above 0.30).

After dimensionality testing, the factor solution was applied to seek evidence of the theoretical model. The first test was performed with the configuration for four domains, according to the original version of the instrument.

Table 3 presents the factor loadings and commonality of the model with four domains. Values equal to or greater than 0.40 for the items were adopted as substantial factor loadings. Acceptable commonality is close to 0.40^(13)^.

**Table 3-:** Factor loadings and commonalities in a model with four domains. São Paulo, SP, Brazil, 2023

Variables	Λ* Factor 1	Λ* Factor 2	Λ* Factor 3	Λ* Factor 4	h2 ^†^
V ^‡^ 1	**0.91**	-0.17	0.02	0.03	**0.69**
V ^‡^ 2	**0.94**	0.02	-0.12	-0.10	**0.72**
V ^‡^ 3	**0.95**	-0.09	0.03	-0.18	**0.73**
V ^‡^ 4	**0.63**	-0.08	0.18	0.27	**0.71**
V ^‡^ 5	**0.88**	-0.02	-0.02	0.04	**0.76**
V ^‡^ 6	**0.81**	0.06	0.04	-0.09	**0.70**
V ^‡^ 7	**0.90**	0.15	-0.07	-0.14	**0.84**
V ^‡^ 8	**0.80**	0.12	-0.04	0.04	**0.77**
V ^‡^ 9	**0.78**	0.08	0.02	0.01	**0.72**
V ^‡^ 10	**0.57**	0.13	0.22	0.09	**0.71**
V ^‡^ 11	**0.60**	0.07	0.06	**0.32**	**0.74**
V ^‡^ 12	**0.68**	-0.02	0.13	0.20	**0.72**
V ^‡^ 13	**0.40**	0.13	0.05	**0.43**	**0.64**
V ^‡^ 14	**0.33**	**0.40**	-0.12	**0.33**	**0.58**
V ^‡^ 15	**0.72**	0.16	0.01	0.18	**0.85**
V ^‡^ 16	**0.67**	0.07	0.14	0.11	**0.73**
V ^‡^ 17	**0.58**	0.15	0.12	0.15	**0.71**
V ^‡^ 18	**0.70**	0.01	0.14	0.06	**0.67**
V ^‡^ 19	**0.75**	0.09	0.01	0.08	**0.73**
V ^‡^ 20	**0.76**	0.05	0.13	0.03	**0.80**
V ^‡^ 21	**0.46**	0.07	**0.32**	0.30	**0.80**
V ^‡^ 22	0.27	0.22	0.25	-0.10	0.37
V ^‡^ 23	0.13	**0.45**	**0.38**	-0.12	**0.64**
V ^‡^ 24	0.08	0.04	**0.86**	-0.04	**0.84**
V ^‡^ 25	-0.06	0.01	**0.90**	0.06	**0.78**
V ^‡^ 26	0.23	0.25	**0.54**	-0.15	**0.71**
V ^‡^ 27	0.01	**0.89**	0.06	-0.01	**0.86**
V ^‡^ 28	0.07	**0.62**	0.09	0.04	**0.53**

*Λ = Factor loading; ^†^h^2^ = Commonality; ^‡^V = Variable

There is no adjustment of the model with four domains, there is a concentration of 20 items in the first domain, and items 11, 13, 14, 21 and 23 have double saturation (measures two factors, considered a technical violation). Item 22 does not present a minimum factor loading of 0.30 and the commonality below 0.40. Therefore, there is no evidence of an adjusted configuration with four domains for the study sample.

Since the exploratory adjustment principle allows and recommends testing different configurations of the instruments, the factor solution was applied with the parameterization with only one dimension. The values of the factor loadings and commonalities are presented in Table 4.

All items presented adequate factor loadings with values above 0.50. Items 22, 25 and 28 presented commonalities below 0.40, but close to the cutoffs. Since the factor loadings of these items were adequate, it was decided to maintain them.

Fitness indexes can be considered to assess the adequacy and quality of a factor solution in EFA. The results presented by the one-dimensional model were considered good, whose indexes: CFI (0.996; 95%CI = 0.994 - 0.998) and NNFI (0.996; 95%CI = 0.994 - 0.998) indicated excellent fit of the model. The RMSEA (RMSEA = 0.040; 95%CI = 0.0275 - 0.0460), GFI (0.991, 95%CI = 0.990 - 0.994) and AGFI (0.991, 95%CI = 0.989 - 0.993) measures were adequate.

The reliability indices presented were: Cronbach’s alpha = 0.97 and McDonald’s omega = 0.97, both above 0.70, indicating adequate reliability^(13)^.

**Table 4-:** Factor loadings and commonalities in a one-dimensional model. São Paulo, SP, Brazil, 2023

**Variables**	**Λ** *	**h2** ^†^
V ^‡^ 1	0.78	0.61
V ^‡^ 2	0.79	0.63
V ^‡^ 3	0.80	0.64
V ^‡^ 4	0.80	0.65
V ^‡^ 5	0.84	0.72
V ^‡^ 6	0.81	0.66
V ^‡^ 7	0.87	0.76
V ^‡^ 8	0.86	0.74
V ^‡^ 9	0.84	0.70
V ^‡^ 10	0.84	0.71
V ^‡^ 11	0.82	0.68
V ^‡^ 12	0.83	0.68
V ^‡^ 13	0.72	0.52
V ^‡^ 14	0.68	0.46
V ^‡^ 15	0.90	0.83
V ^‡^ 16	0.85	0.73
V ^‡^ 17	0.84	0.71
V ^‡^ 18	0.81	0.67
V ^‡^ 19	0.84	0.71
V ^‡^ 20	0.89	0.80
V ^‡^ 21	0.86	0.74
V ^‡^ 22	0.56	0.31
V ^‡^ 23	0.68	0.46
V ^‡^ 24	0.68	0.47
V ^‡^ 25	0.61	0.37
V ^‡^ 26	0.72	0.52
V ^‡^ 27	0.70	0.49
V ^‡^ 28	0.61	0.37

*Λ = Factor loading; ^†^h^2^ = Commonality; ^‡^V = Variable

## Discussion

This study reported the process of translating and adapting the AICLS from English to the Brazilian context. Although it is recognized that this is a new scale, only one Japanese study was identified that translated and cross-culturally adapted the AICLS, in order to enable comparison of the scale in question^(27)^.

The CCA was guided by recommendations for cross-cultural adaptation of measurement instruments^(10)^, which are widely used in this type of study, with international acceptance of this methodology^(28)^. CCA requires great methodological rigor, and it is essential that the values reflected in each item are equivalent between one culture and another^(29)^.

Semantic and cultural equivalences must be considered in the CCA process. The discrepancies found in the versions presented by the translators were resolved in the synthesis of the translations and subsequently sent to the back-translation phase, which is recommended as an indicator of the quality of psychometric evidence, and allows the identification of possible discrepancies^(28)^ and the highlighting of inconsistencies or conceptual errors in the translation^(30)^. The reconciled version did not present misinterpretations of the items, as confirmed by the author of the original instrument.

In this context, the quality of the translation/adaptation process is crucial to ensure the validity and usefulness of the adapted test, through a rigorous process whose objective is to maintain the equivalence of content and cultural meaning between the original and the translated/adapted test and to promote the comparability of scores among participants from different cultural groups^(31)^.

The analysis of the validity evidence related to the content of the Brazilian version of the EALCI was carried out in stages. For content validation, a committee of interprofessional experts, with expertise in leadership and collaborative interprofessional practice, clarified the discrepancies and suggested adjustments for better understanding. In designing a coherent instrument for the population it will be aimed at, this phase is essential^(32)^.

Unlike the original instrument, in which the authors reported that only one item was revised in terms of its wording, the Brazilian version had 12 items that required revision and required a second round with the committee of experts. After this second assessment, the items had their content validated. In the original version, the authors used the Content Validity Index (CVI), however, this can inflate the results by combining responses with scores of 3 and 4 from the experts’ analysis^(33)^. In this study, the CVR calculation was used, a more robust method for establishing and quantifying content validity^(14)^ and which adapts the agreement to the number of experts, ensuring the quality of the assessment^(26)^.

During the pilot test, which followed the recommendations in the literature^(10)^ and aimed to verify whether the items, instructions and response scale were understandable, especially by those for whom the instrument was intended^(30)^, it was identified that item 7 was the least understood by the respondents, due to an expression that can be considered common in the academic environment, but which did not have any relevance to the target audience and which had to be removed (*status quo*).

In the validation of the internal structure, necessary to verify whether the measurement attributes correspond to the theoretical attributes^(21)^ and to adjust the model, multiple tests were used, in accordance with contemporary recommendations for evidence of validity^(12)^.

It was also analyzed whether the factor structure was adequately represented by its dimensionality^(21)^. In the Brazilian version, the four-dimensional model was not reproduced. The divergence found in the parallel analysis indicated additional testing of dimensionality, due to recommendations in the literature that dimensionality analysis should not be limited to one technique^(34)^. Unidimensionality remained and in the EFA, the unidimensional model was the most appropriate, and did not indicate the maintenance of the dimensions in the population of this study. It is worth mentioning that in the analyses of this study, robust standards were followed, described in contemporary psychometrics, analyses considered to be of excellence in the development and adaptation of instruments^(11-12)^.

In the analysis of the original instrument^(5)^, the authors preliminarily indicated that the Leadership Ability dimension would probably not be maintained in the final model, since several items indicated that they assessed a small aspect of the construct. The sample of the original study did not reach the minimum number indicated in the literature, with only 101 professionals, an aspect that may have impacted the results of the analyses performed and that explains the differences between the two studies. Another difference is that they did not perform dimensionality analyses, EFA and CFA tests, and opted for Cronbach’s alpha testing, considered in this study to be insufficient for analyzing the adequacy of the original model.

Therefore, in this study, different analysis techniques were chosen from those used in the original model, since it recognizes the existence of more modern techniques indicated in the specialized literature, and that despite the choice to follow analysis processes different from the original instrument, the EALCI was considered a fundamental instrument, with contributions to reality and to the Brazilian context considering its theoretical framework, broad tradition and application in the practice of interprofessionality constructs^(2)^.

It is important to highlight that the Japanese version^(27)^ did not have the same dimensionality as the original AICLS instrument. After exploratory factor analysis using the 28 items, divided into four factors, the instrument was reduced to ten items, distributed in three dimensions. The final instrument, called AICLS-J, was applied to a sample of 675 participants.

Different cultures may understand, experience or express the same construct in different ways, and this implies grouping items in dimensions different from those observed in the original version. Regarding interprofessionality, two instruments did not reproduce the dimensionality of the original instrument in the Brazilian context. In the adaptation of the Readiness for Interprofessional Learning Scale (RIPLS) instrument, the original four-factor structure was not reproduced equally to the original model, and the factor analysis indicated a version with 27 items distributed in three dimensions: teamwork and collaboration, professional identity and patient-centered health care^(35)^. The other instrument for translation and cross-cultural adaptation, the Interprofessional Collaboration Measurement Scale (IPC), did not replicate the three factors of the original version in the exploratory and confirmatory factor analyses in the Brazilian sample, and indicated that it was a one-dimensional instrument^(36)^, similar to that found in this study.

In both cases, the difficulty in reproducing the same dimensions of the original version can be attributed to cultural differences, educational contexts and distinct practices, since they are constructs with multiple interdependent aspects. Given this assumption, this can occur not only between countries, but also between regions of the same country, between different types of health services. There is agreement that differences in dimensionality should not be considered a limitation, but may reflect that in the research application scenario the construct was perceived as a holistic construct, or even indicate gaps in interprofessional training and practice in health^(36)^. Therefore, the guidelines of the area are reinforced and corroborated, that there must be an analysis of the equivalences of internal structure and the measurement of the scale considering regional differences^(12)^ and that this analysis must be carried out when choosing to use a measurement scale.

Thus, it can be inferred that interprofessional collaborative leadership can be influenced by aspects of the organizational structure, power relations, professional training and institutional culture that vary significantly between countries, regions and types of services. The adaptation process allows the instrument to be sensitive to the linguistic, cultural and social specificities of its context, maintain the conceptual and theoretical foundations of the original instrument, and contribute to the advancement of research and the applicability of new management models, based on the interprofessional organization of teams.

In the analysis of evidence of reliability of the Brazilian unidimensional version of the EALCI, McDonald’s omega coefficient and Cronbach’s alpha were higher than 0.70. The Japanese version used only Cronbach’s alpha, with a value = 0.987^(27)^. In the present study, it was decided to demonstrate two indicators to increase the accuracy of the interpretation.

When comparing the context of the items of the CSLS instrument^(8)^ with the Brazilian version of the EALCI, although the hierarchical structure is not assessed, there is a similarity between the theoretical contributions of the two instruments and between the items referring to collaboration between team members, with their well-established roles, encouraging creativity and discussions in care planning, as well as sharing of knowledge, skills and expertise, according to the needs of each situation.

Thus, the instrument translated and validated into Portuguese corroborates the idea that collaborative leadership is fundamental and prepares leaders of interprofessional teams to coordinate and collaborate in teams, respecting the patient and family regarding their own care decisions, as the path to offering quality and safe health care. Working in an interprofessional and collaborative manner requires everyone to be jointly responsible for the results produced^(5)^.

Having an instrument available in national and international literature capable of adequately measuring the construct of interprofessional collaborative leadership is an advance in the field of interprofessionality. It can even be used in training and professional development programs that aim to address the development of this skill, especially from the perspective of involving patients and caregivers in decision-making processes, with a view to improving the patient experience and the quality and safety of care, a practice that is not yet fully consolidated in health services^(37)^.

The limitations of this study include the fact that there are few analyses of the instrument translated into other languages and that the Canadian version itself had a smaller sample than indicated in the literature. Only the Japanese study was identified^(27)^, and this study attempts to fill the gap identified, with future indications of new applications and analyses of the EALCI. There are no other instruments that measure interprofessional collaborative leadership or other similar scales validated in Brazil, which does not allow comparisons.

Although the dimensionality of the original scale was not replicated, an aspect that can be considered a limitation, it is worth noting that more contemporary and different analysis tests were used, as they were considered more relevant in the present study for this type of scale. It is worth noting that among the responsibilities of the researchers is to present the results with respect to transparency and the ethical principles that govern scientific practice, even if they were not in line with those of the original studies. These results also contribute to the advancement of knowledge and to the improvement of the scale that measures interprofessional collaborative leadership. Thus, the limitations found should be considered in future research and in the improvement of the EALCI.

Another limitation is that the interactions between the professionals who made up the teams in the areas of activity in the study were not analyzed, an aspect that could configure an analysis of the criterion validity. Thus, there are indications for further research, both on the application of the translated and validated instrument in other contexts of interprofessional practice, as well as convergent validity^(38)^, to establish correlation of the instrument with instruments that assess similar constructs, especially with already validated and widely used scales of interprofessional collaborative practice.

As implications for practice, it is indicated that the EALCI can be used in the Brazilian context for the global measurement of interprofessional collaborative leadership, and contemporary analysis procedures are indicated in different contexts of health care, to strengthen its applicability.

## Conclusion

The EALCI, composed of 28 items, has proven to be an instrument for measuring interprofessional collaborative leadership, with a unidimensional structure and has presented adequate evidence of content validity, response process, internal structure and reliability. It is a valid scale for making situational diagnoses, supporting institutional intervention policies and the impact of educational programs from the perspective of interprofessional practice.

Collaborative leadership is an indispensable skill in interprofessional teamwork, considered essential in health care to ensure the centrality of the patient and family. The provision of this scale will contribute to the understanding of the processes involved in this form of leadership, its occurrence in interprofessional teams, and can assist health institutions in professional development programs and contribute to the implementation of this care model.

## Data Availability

Datasets related to this article will be available upon request to the corresponding author.
