# Supplementary material for: Validity evidence of the Brazilian version of the Assessment Interprofessional Collaborative Leadership Scale
Source: Rev Lat Am Enfermagem. 2025 Nov 17;33:e4719. doi: 10.1590/1518-8345.7894.4719 (PMC12628366; doi:10.1590/1518-8345.7894.4719)
Supplement: Translation

# Translation: Evidências de validade da versão brasileira da *Assessment Interprofessional Collaborative Leadership Scale* *

**DOI:** 10.1590/1518-8345.7894.4720

**Authors:** Natalia Dalforno da Silva, Geisa Colebrusco de Souza Gonçalves, Jaqueline Alcantara Marcelino da Silva, Flávio Rebustini, Alexandre Pazetto Balsanelli

**Affiliations:** 1 Universidade Federal de São Paulo, Escola Paulista de Enfermagem, São Paulo, SP, Brasil.; 2 Hospital Sírio Libanês, São Paulo, SP, Brasil.; 3 Universidade Federal de São Carlos, Departamento de Enfermagem, São Carlos, SP, Brasil.; 4 Universidade de São Paulo, Escola de Ciências, Humanidades e Artes, São Paulo, SP, Brasil.

**Keywords:** Liderança, Equipe de Assistência ao Paciente, Relações Interprofissionais, Estudo de Validação, Profissionais de Saúde, Gestão de Recursos da Equipe de Assistência à Saúde

## Abstract

buscar evidências de validade da *Assessment Interprofessional Collaborative Leadership Scale* para o português – Brasil e analisar evidências de conteúdo, processo de resposta e estrutura interna.

estudo de avaliação psicométrica, com tradução, retrotradução e avaliação por comitê de especialistas acerca da equivalência linguística e de conteúdo. O pré-teste foi realizado com 40 participantes e, posteriormente, a versão final foi aplicada com 318 trabalhadores da saúde. Realizou-se a análise fatorial exploratória e o teste de confiabilidade por meio do alfa de Cronbach e ômega de McDonald.

na validação de conteúdo, quatro itens apresentaram pontuações abaixo do valor crítico. Dos participantes, a maioria era da enfermagem (81%) e fisioterapeutas (10%), do sexo feminino (79%). A fatorabilidade obtida foi Kaiser-Meyer-Olkin = 0,94 e Esfericidade de Bartlett = 2991,4 com p < 0,05. A análise paralela indicou modelo unidimensional, com variância explicada por 63,71%. Índices de adequação do modelo apresentaram níveis satisfatórios e índices de confiabilidade adequados (alfa de Cronbach = 0,97 e ômega de McDonald = 0,97).

a Escala de Avaliação da Liderança Colaborativa Interprofissional, de estrutura unidimensional, com 28 itens, apresentou boas evidências de validade de conteúdo, processo de resposta e estrutura interna considerada adequada para mensurar liderança colaborativa interprofissional.

## Destaques

**(1)** Instrumento para medir liderança colaborativa interprofissional. **(2)** Instrumento para implementar modelo assistencial de cuidado interprofissional. **(3)** Diagnóstico para fundamentar programas de desenvolvimento interprofissional. **(4)** Evidências de validade de instrumento de liderança colaborativa interprofissional.

## Introdução

A prática interprofissional colaborativa é uma forma de organização do trabalho, imprescindível para abordar questões de saúde complexas e proporcionar atendimento integral^(1)^. Ela requer esforços coordenados para garantir o compartilhamento de informações e decisões para que a *expertise* de cada membro da equipe seja viabilizada na implementação dos cuidados e no atendimento das necessidades de saúde^(2)^.

Dentre as competências da prática interprofissional colaborativa está a liderança colaborativa, na qual profissionais trabalham em conjunto com pacientes e famílias para promover a tomada de decisões compartilhadas e produzir corresponsabilidades na equipe^(2)^.

Diferentemente da liderança tradicional, hierárquica e centralizada, a liderança colaborativa interprofissional estabelece-se no compartilhamento de responsabilidades, construção coletiva e articulada de soluções a partir de múltiplas perspectivas, de profissionais e pacientes, nas decisões clínicas para ampliar a qualidade da assistência^(3)^. Também descrita na literatura como liderança coletiva ou distributiva, é considerada estratégia para implementar intervenções complexas, promover comunicação e participação segura, envolver as partes interessadas, num ambiente em que se busca a equidade^(4)^. Contudo, sua aplicação prática no cotidiano dos serviços segue pouco explorada.

Nesse cenário, o instrumento *Assessment Interprofessional Collaborative Leadership Scale* (AICLS)^(5)^ foi construído e publicado no contexto canadense em 2019, com a proposta de medir a liderança colaborativa interprofissional nas instituições de saúde, por meio das percepções individuais dos profissionais de saúde que compõem as equipes^(5)^. A gênese de sua construção é o modelo teórico que explora a inter-relação entre estruturas verticais e formais na instituição e equipes colaborativas, como novas possibilidades do exercício de liderança^(6)^.

Embora os contextos de formação e de prática em saúde no Brasil e no Canadá apresentem especificidades locais, a literatura sobre trabalho em equipe interprofissional tem estabelecido amplamente as competências interprofissionais necessárias, como comunicação, colaboração e a liderança colaborativa, em diversos cenários de saúde^(7)^.

Estudos específicos sobre liderança colaborativa interprofissional são escassos, logo, identifica-se uma lacuna nos instrumentos de medida. Reconhece-se a existência da *Clinical Shared Leadership Scale* (CSLS)^(8)^, contudo esta não avalia a tomada de decisão compartilhada com envolvimento direto de pacientes e cuidadores – característica fundamental identificada nessa competência – conforme se encontra na pesquisa com a participação significativa de jovens na proposição e desenvolvimento de cuidados integrados, baseado na liderança colaborativa interprofissional^(9)^. Assim, a liderança colaborativa interprofissional requer a participação ativa de pacientes e família (usuários do serviço), razão pela qual optou-se pela tradução do AICLS^(5)^, dado seu avanço e relevância teórica para a realidade e o contexto do sistema de saúde brasileiro.

O instrumento AICLS, no Canadá, passou pela análise de validade de conteúdo e foi considerado altamente relevante em sua versão preliminar, sua evidência de confiabilidade foi de 0,96, e suas dimensões variaram de 0,85 a 0,92 [Relação simbiótica = 0,87; Atenção plena (*Mindfulness*) = 0,92; Recursos compartilhados = 0,92 e Capacidade de liderar = 0,85]^(5)^.

Assim, torna-se relevante a tradução e validação deste instrumento com a premissa de ser uma medida eficaz para análise da liderança colaborativa em equipes interprofissionais e que pode impactar na compreensão dessa competência para alcance da melhoria da qualidade e da segurança do cuidado ofertado pelas equipes. Nesse sentido, o objetivo deste estudo foi buscar evidências de validade da *Assessment Interprofessional Collaborative Leadership Scale* (AICLS) para o português - Brasil e analisar evidências de conteúdo, processo de resposta e estrutura interna.

## Método

### Tipo do estudo

Trata-se de um estudo de avaliação psicométrica, cujas evidências de validade e adaptação transcultural (ATC)^(10)^ se deram por meio das fases: tradução; síntese, retrotradução, avaliação por comitê de especialistas, pré-teste do instrumento e, por fim, análise das evidências de validade do instrumento^(10-11)^. O estudo seguiu as diretrizes contempladas no *Test Adaptation Reporting Standards* (TARES)^(12)^.

A tradução foi realizada por dois tradutores independentes, com elaboração de uma versão síntese única, por terceiro tradutor^(10)^, avaliada e aprovada pela autora do instrumento.

A equivalência da versão em português foi estabelecida a partir de quatro dimensões: semântica, idiomática, cultural e conceitual por 17 especialistas em prática interprofissional colaborativa e/ou traduções de instrumentos de medida. Após a aprovação pelo comitê de especialistas, seguiu-se para o teste-piloto com 40 trabalhadores assistenciais ou de gestão, que compunham equipes de unidades de internação de um hospital.

Para a avaliação das propriedades psicométricas, uso e adoção do instrumento AICLS^(5)^ no Brasil, buscou-se reunir evidências de validade baseadas no conteúdo, processo de resposta, estrutura interna e verificação da confiabilidade.

A validação de conteúdo é composta por avaliação qualitativa (comitê de especialistas) e avaliação quantitativa de cada item, ao se considerar a importância individual do item na composição do instrumento e representação do construto^(13)^, por meio do cálculo da *Content Validity Ratio* (CVR)^(14)^.

Na validação do processo de resposta, verifica-se como o item é interpretado e sua relação com o construto medido^(11)^. Participantes do teste-piloto analisaram a viabilidade, compreensibilidade e facilidade do uso do instrumento. Houve registro do tempo de resposta, dado que pode trazer evidências da complexidade dos processos de resposta^(11,15-16)^.

A análise da estrutura interna do construto foi realizada por Análise Fatorial Exploratória (AFE), que avalia, por meio das cargas fatoriais, a pertinência de cada item para o fator^(16)^. A retenção de fatores influencia substancialmente os resultados da AFE, como também tem fortes implicações teóricas, pois predetermina a dimensionalidade assumida de um construto latente. Devido à sua natureza exploratória, a AFE é utilizada em casos cuja compreensão ainda é incipiente acerca da estrutura fatorial do conceito latente^(17)^, como é o caso da AICLS, recém descrita na literatura^(5)^. Índices de ajuste da solução fatorial foram utilizados para apoiar na determinação do número de fatores retidos^(17)^, e na adequação da estrutura fatorial encontrada^(18)^.

A confiabilidade também foi verificada, utilizando dois indicadores, conforme recomendação da literatura, para aumentar a confiabilidade da interpretação^(19)^.

### Local

O estudo foi desenvolvido em um hospital geral privado, de nível terciário e grande porte, localizado no município de São Paulo (SP), Brasil, considerado um centro de referência internacional em saúde.

### População e amostra do estudo

A população do estudo foi composta por trabalhadores de saúde, em cargos gerenciais e assistenciais, que compunham equipes interprofissionais de unidades críticas e unidades de internação, clínicas e cirúrgicas.

O tamanho da amostra foi calculado com base nas recomendações de verificação da estrutura interna de instrumentos, a qual indica um número mínimo de 300 participantes^(13)^. Neste estudo, alcançou-se uma amostra por conveniência de 318 trabalhadores de saúde que compunham equipes, enfermeiros, técnicos de enfermagem, fisioterapeutas, médicos, farmacêuticos, psicólogos e nutricionistas.

O critério de inclusão foi trabalhadores atuantes há, no mínimo, um ano na instituição, em cargos de gestão ou assistência, com atividades compartilhadas com pelo menos um trabalhador de outra área de formação. O critério de exclusão utilizado foi trabalhadores que estavam afastados no momento da coleta de dados.

### Variáveis do estudo

O AICLS contempla 28 itens, divididos em quatro dimensões: Relação simbiótica; Atenção plena (*Mindfulness*); Recursos compartilhados; e Capacidade de liderar^(5)^.

A dimensão Relação simbiótica, composta por cinco itens, é definida por uma colaboração na qual todos os membros da equipe têm seus papéis bem estabelecidos e se adaptam reciprocamente às demandas que surgem^(5)^.

A dimensão Atenção plena (*Mindfulness*) é formada por nove itens, caracterizada por foco atento e estendido, com atenção às situações e experiências imediatas à medida que elas aparecem^(5)^.

A dimensão Recursos compartilhados é composta por sete itens, definida por um ambiente que incentiva a abertura, entre os profissionais, para compartilhar conhecimento, habilidade e *expertise* dentro de uma equipe^(5)^.

A dimensão Capacidade de liderar, composta por sete itens, é definida por uma vontade de liderar e aceitar a responsabilização para o cargo de liderança^(5)^.

Os respondentes indicam seu nível geral de concordância com a ocorrência de cada item em uma escala de classificação do tipo *Likert* de cinco pontos: 1 - Nunca; 2 - Raramente; 3 -Ocasionalmente; 4 - Na maioria das vezes; e, 5 - Sempre. As classificações produzem pontuações de 28 a 140^(5)^.

### Coleta de dados

A versão produzida após as etapas de validação de conteúdo e processo de resposta foi transformada em questionário eletrônico, junto a questões de caracterização da amostra. Os dados foram coletados por meio da plataforma *Research Electronic Data Capture* (RedCap)^(20)^. O convite para a participação foi enviado via correio eletrônico para as coordenadoras de área, que encaminharam aos profissionais/trabalhadores de saúde de suas respectivas equipes.

### Período

O período de coleta de dados ocorreu entre 15 de julho a 15 de setembro de 2023.

### Tratamento e análise dos dados

Para iniciar a AFE e verificar a fatorabilidade dos dados, foi calculado o índice Kaiser-Meyer-Olkin (KMO) e realizado o Teste de Esfericidade de Bartlett (TEB)^(13)^.

Para a extração de fatores, utilizou-se o *Unweighted Least Squares* (ULS), com rotação *oblimin*, a partir de uma matriz de correlação policórica^(13)^ e, posteriormente, foi explorada a dimensionalidade do instrumento, por meio da técnica de análise paralela, *Optimal Implementation of Parallel Analysis* (PA), abordagem recomendada para um conjunto de variáveis ordenadas com pontuação politômica^(21)^, método de Hull com *Comparative Fit Index* ^(22)^ e aplicação da *Closeness of dimensionality* ^(23)^ *,*composta por três indicadores: *Item Unidimensional Congruence* (I-UniCo), *Item Explained Common Variance* (I-ECV) e *Mean of Item Residual Absolute Loadings* (MIREAL). Valores de I-UNICO > 0,95, I-ECV > 0,85 e MIREAL < 0,30 sugerem que os dados podem ser tratados como essencialmente unidimensionais^(23)^.

A solução fatorial foi aplicada via modelagem por equações estruturais e, para a verificação de necessidades de ajustes, foram calculados os índices: *Root Mean Square Error of Approximation* (RMSEA - Raiz do Erro Quadrático Médio de Aproximação); *Non-Normed Fit Index* (NNFI - Índice de Ajuste Não Normatizado); CFI (Índice de Ajuste Comparativo); *Goodness of Fit Index* (GFI - Índice de Qualidade do Ajuste); *Adjusted Goodness of Fit Index without diagonal values* (AGFI - Índice Ajustado de Qualidade do Ajuste)^(24)^. A confiabilidade foi verificada utilizando os índices alfa de Cronbach e ômega de McDonald^(19)^.

Todas as análises foram realizadas no *software Factor*.

### Aspectos éticos

Os aspectos éticos da pesquisa foram seguidos conforme a Resolução n^o^. 466 de 2012^(25)^, tanto na fase de avaliação do comitê de especialistas quanto na aplicação do teste-piloto e pré-teste. As etapas aconteceram após a aprovação do Comitê de Ética em Pesquisa (CEP) da Universidade Federal de São Paulo e da instituição coparticipante, hospital onde foi realizada a pesquisa, sob Pareceres n^o^. 5.533.152 e n^o^. 5.571.337, respectivamente.

## Resultados

### Adaptação transcultural e validação de conteúdo

A análise de equivalência resultou em 62,5% dos 28 itens com concordância maior que 80%. Após a primeira rodada da análise do comitê de especialistas, 12 itens que tiveram a taxa de concordância menor que 0,80 passaram por revisão, assim como o nome do instrumento, as duas primeiras frases explicativas de introdução e o parágrafo final.

As sugestões foram acatadas e, após as modificações, realizou-se uma nova rodada com o comitê de especialistas para a revisão dos itens e a escolha, entre duas opções, da melhor alternativa, levando em consideração seus conceitos e significados dentro da cultura brasileira. A definição final dos 12 itens está descrita na Figura 1.

**Figura 1-:** Modificações realizadas pelos autores nos itens após sugestões e segunda rodada do comitê de especialistas. São Paulo, SP, Brasil, 2022

**Item após modificação**	**Modificação**
Seção 1: RELAÇÃO SIMBIÓTICA: Relação simbiótica é uma colaboração na qual todos os membros da equipe têm seus próprios papéis bem estabelecidos e se adaptam mutuamente à dinâmica de mudanças de demandas.	Trocada palavra “ambos” por “todos” e alterada a posição da palavra “dinâmica”
2. Incentiva os membros da equipe a valorizar a expertise uns dos outros.	Trocada palavra “competências” por “expertise”
3. Incentiva os membros da equipe a aproximar suas capacidades complementares (compartilhando conhecimentos, habilidades e experiências) para direcionar o plano de cuidados.	Trocada palavra “explorar” por “aproximar”, invertida ordem das palavras “compartilhando conhecimentos”, e trocado “abordar o planejamento” por “direcionar o plano”.
Seção 2: ATENÇÃO PLENA ( *Mindfulness* ): Atenção Plena é o foco consciente e prolongado da atenção em experiências imediatas à medida que elas acontecem.	Acrescentado o termo “ *Mindfulness* ” e trocada palavra “intencional” por “consciente”
7. Incentiva os membros da equipe a se concentrarem além do *status quo* (isto é, a maneira usual de fazer as coisas) em questões relevantes e essenciais do cuidado.	Trocado “essência” por “essenciais”
8. Incentiva os membros a considerarem soluções criativas para o planejamento de cuidados complexos a pacientes/clientes.	Trocado “atendimentos mais” por “cuidados”
11. É receptivo para apoiar as mudanças sugeridas pelos membros da equipe.	Invertida a ordem da palavra “apoio”.
16. Incentiva os membros da equipe a estabelecerem metas compartilhadas para o trabalho em equipe.	Trocado “sobre seu” por “para o”
21. Compartilha o trabalho entre os membros da equipe, de acordo com suas capacidades, quando os planos de cuidado são implementados	Invertida a ordem das palavras no início da frase
22. Os membros da equipe apoiam que os pacientes/clientes sejam líderes colaborativos	Trocado “apoiem” por “apoiam”
24. Todos os membros da equipe aceitam e se responsabilizam pelo seu trabalho compartilhado em equipe	Trocado “responsabilidade” por “se responsabilizam”
27. Há apoio para o rodízio do líder da equipe de acordo com as necessidades do nosso planejamento do cuidado.	Trocado “apoiemos” por “há apoio” e “conforme” por “de acordo”.

Na validação de conteúdo quantitativa, o valor crítico do CVR aceitável para 17 especialistas foi igual a 0,529^(26)^ e indicou a aderência do item no estudo. Quatro itens obtiveram pontuações abaixo de 0,529 em alguma das categorias de avaliação: relevantes teoricamente, pertinentes na prática, suficientemente abrangentes e compreensíveis no conjunto e dimensionalidade.

O item 2, na seção 1, “Incentiva os membros da equipe a se concentrarem além do *status quo* (isto é, a maneira usual de fazer as coisas) em questões relevantes e essenciais do cuidado”, apresentou pontuação abaixo de 0,529 quando avaliada sua relevância teórica, pertinência prática e abrangência.

O item 19, da seção 3, “O processo de tomada de decisão se concentra em objetivos compartilhados de todos os membros da equipe”, apresentou pontuação menor na avaliação de abrangência.

Já na seção 4, dois itens obtiveram pontuações inferiores a 0,529. O item 22 “Os membros da equipe apoiam que os pacientes/clientes sejam líderes colaborativos” foi avaliado com menor pontuação em pertinência prática, abrangência e dimensionalidade, e o item 23 “Os membros da equipe estejam dispostos a assumirem a função de liderança de equipe quando solicitados”, em abrangência e dimensionalidade. Todos os itens foram mantidos no instrumento neste momento, já que apresentaram valores abaixo da razão considerada em alguma das categorias avaliadas e não em sua totalidade.

### Validação do processo de resposta

Quanto à validade do processo de resposta, realizada por meio de entrevista, no teste-piloto, certificou-se a viabilidade do instrumento para uso. Os participantes utilizaram, em média, nove minutos e 27 segundos na resposta do instrumento. O desvio-padrão foi de três minutos e quinze segundos e o tempo máximo de resposta foi de 15 minutos e 51 segundos.

Os respondentes apresentaram dificuldade com o termo “*status quo*” no item 7, na seção 2, apesar de a explicação estar em parênteses dentro do próprio item, conforme indicado pelo comitê de especialistas. Como sugestão, foi retirado o termo “*status quo”,* e esse item ficou definido como”Incentiva os membros da equipe a desenvolverem processos que levem à criação de um ambiente onde tomadas de decisões são compartilhadas”.

A compreensão das opções de respostas dos itens foi adequada para 100% dos participantes, assim como a organização geral dos itens. Na avaliação da abrangência dos itens, 87,5% dos participantes consideraram adequada, e um deles sugeriu a retirada de itens que julgava parecidos.

Quanto à dificuldade de preenchimento do instrumento, 62,5% dos participantes relataram não haver dificuldades e 20 sugestões foram relatadas e consideradas. Assim, oito itens foram ajustados (itens 1 e 3 da seção 1; itens 7, 9 e 10 na seção 2; itens 19 e 21 na seção 3; e item 25 na seção 4).

A versão final do instrumento após as alterações sugeridas foi respondida por 318 participantes, sendo que seis assinalaram a opção de estar há menos de um ano na instituição, o que configuraria sua exclusão após a coleta de dados, porém, pela pequena porcentagem (1,8%), em relação ao total da amostra, optou-se pela manutenção na apresentação dos resultados.

Quanto às características sociodemográficas, destaca-se que, majoritariamente, a amostra foi composta por pessoas do sexo feminino (79%) e na faixa etária de 31-40 anos (44%). A carga horária de trabalho semanal de 30-40 horas foi prevalente (67%). Quanto à maior titulação, 53% dos participantes tinham especialização. Os enfermeiros (44%), seguidos dos técnicos de enfermagem (37%) e fisioterapeutas (10%), foram os profissionais com maior participação. Sobre o tempo de atuação profissional, o intervalo de 11 a 15 anos foi predominante (24%) e de atuação na unidade foi de um a três (31% dos participantes). Sobre o tipo de unidade de atuação, os participantes atuavam na Unidade Semi-Intensiva (26%), seguida da Unidade de Terapia Intensiva (21%).

### Validação da estrutura interna

Procedeu-se à AFE com base na matriz policórica e foi verificado se os itens eram fatoráveis por meio da *Measure of Sample Adequacy* (MSA). Na modelagem inicial utilizando os 28 itens do instrumento, bons índices de adequação foram encontrados, sendo TEB: 2991,4 (gl = 378; P = 0,000010) e KMO: 0,94977 com *bootstrap* 95% Intervalo de Confiança (IC) do KMO = (0,573 - 1,204).

A primeira análise no estudo da dimensionalidade/fatores do instrumento foi realizada pela análise paralela (AP), que indicou a existência de apenas uma dimensão, com variância explicada de 63,71% da variável latente. Pela divergência entre o modelo original e o obtido pela AP, optou-se por testagens adicionais da dimensionalidade.

A segunda técnica utilizada foi a Hull com o CFI, demonstrada na Tabela 1.

Novamente, o resultado apontou apenas uma dimensão. O terceiro teste adotado foi a técnica de unidimensionalidade/multidimensionalidade, com os índices UniCo, ECV e REAL demonstrados na Tabela 2.

**Tabela 1-:** Dimensionalidade pela Hull com *Comparative Fit Index*. São Paulo, SP, Brasil, 2023

**Número fatores**	**CFI** *	**Graus de liberdade**	***Scree test***
0	0,00	378	0,00
1	0,99	350	**250,61** *
2	1,00	323	0,00
3	1,00	297	

*CFI = *Comparative Fit Index*

**Tabela 2-:** *Closeness of dimensionality*. São Paulo, SP, Brasil, 2023

**Variáveis**		**I-UniCo** *	**I-ECV** ^†^	**I-REAL** ^‡^
V ^§^ 1. Ajuda os membros a valorizarem suas contribuições para o trabalho em equipe.		0,99	0,93	0,22
V ^§^ 2. Incentiva os membros da equipe a valorizar a expertise uns dos outros.		0,99	0,92	0,23
V ^§^ 3. Incentiva os membros da equipe a aproximar suas capacidades complementares (conhecimentos, habilidades e experiências compartilhadas) para direcionar o plano de cuidados.		1,00	0,97	0,14
V ^§^ 4. Permite que todos os membros da equipe tenham uma oportunidade de expressar suas opiniões.		1,00	0,98	0,11
V ^§^ 5. Possibilita que os membros da equipe vejam seus resultados compartilhados como significativos e valiosos.		0,99	0,95	0,20
V ^§^ 6. Incentiva os membros da equipe a desenvolverem processos que levem à criação de um ambiente onde tomadas de decisões são compartilhadas.		1,00	0,99	0,08
V ^§^ 7. Incentiva os membros da equipe a se concentrarem para além da maneira usual de fazer as coisas, em questões relevantes e essenciais do cuidado.		1,00	0,98	0,13
V ^§^ 8. Incentiva os membros a considerarem soluções criativas para o planejamento de cuidados complexos a pacientes/ clientes.		0,99	0,97	0,16
V ^§^ 9. Incentiva os membros da equipe a reavaliar maneiras tradicionais de lidar com situações semelhantes.		1,00	0,98	0,11
V ^§^ 10. Incentiva discussões abertas entre os membros da equipe acerca de questões do planejamento do cuidado.		1,00	0,99	0,04
V ^§^ 11. É receptivo para apoiar as mudanças sugeridas pelos membros da equipe.		0,99	0,97	0,15
V ^§^ 12. Incentiva os membros da equipe a se adaptarem a diversas situações.		1,00	0,98	0,11
V ^§^ 13. Incentiva os membros da equipe a questionarem algo que não faça sentido.		1,00	0,97	0,12
V ^§^ 14. Apoia a inovação criativa dos membros da equipe em situações em que há incertezas no planejamento do cuidado ao paciente/cliente.		1,00	0,98	0,10
V ^§^ 15. Se certifica de que todos os membros da equipe tenham a oportunidade de compartilhar suas perspectivas acerca do planejamento do cuidado ao paciente/cliente.		1,00	0,98	0,13
V ^§^ 16. Incentiva os membros da equipe a estabelecerem metas compartilhadas para o trabalho em equipe.		1,00	0,99	0,05
V ^§^ 17. Facilita ajustes das funções dos membros da equipe às necessidades situacionais.		1,00	0,99	0,03
V ^§^ 18. Incentiva os membros da equipe a assumirem a responsabilidade de suas contribuições no processo de tomada de decisões da equipe.		1,00	0,99	0,06
V ^§^ 19. Se concentra em objetivos compartilhados, de todos os membros da equipe, no processo de tomada de decisão.		1,00	0,97	0,14
V ^§^ 20. Incentiva a integração de perspectivas visando facilitar processos compartilhados de tomada de decisão no desenvolvimento do plano de cuidado do paciente/cliente.		1,00	0,99	0,06
V ^§^ 21. Compartilha o trabalho entre os membros da equipe, de acordo com suas capacidades, quando os planos de cuidados são implementados.		1,00	0,99	0,05
V ^§^ 22. Os membros da equipe apoiam que os pacientes/clientes sejam líderes colaborativos.		0,98	0,83	0,26
V ^§^ 23. Os membros da equipe estejam dispostos a assumirem a função de liderança da equipe quando solicitados.		0,93	0,72	0,43
V ^§^ 24. Todos os membros da equipe aceitam e se responsabilizam pelo seu trabalho compartilhado em equipe.		0,86	0,62	0,54
V ^§^ 25. Todos os membros da equipe contribuam para objetivos comuns e compartilhados.		0,76	0,54	0,58
V ^§^ 26. Os membros da equipe orientem uns aos outros para que todos sejam capazes de liderar a equipe de forma eficaz.		0,94	0,74	0,43
V ^§^ 27. Há apoio para o rodízio do líder da equipe, de acordo com as necessidades do nosso planejamento de cuidado.		0,98	0,85	0,30
V ^§^ 28. Selecionamos juntos o líder da nossa equipe.		0,99	0,88	0,23
Avaliação	Geral			
UniCo ^||^	=	0,98		
ECV ^¶^	=	0,92		
MIREAL ^**^	=	0,19		

*I-UniCo = *Item Unidimensional Congruence*; ^†^I-ECV - Item *Explained Common Variance*; ^‡^I-REAL = *Item Residual Absolute Loadings*; ^§^V = Variável; ^||^UniCo = *Unidimensional Congruence*; ^¶^ECV = *Explained Common Variance*; ^**^MIREAL = *Mean of Item Residual Absolute Loadings*

A partir do resultado apresentado, o modelo geral pode ser considerado, novamente, unidimensional, cujos valores dos índices dos itens 23, 24, 25 e 26 não sustentam a unidimensionalidade (I-REAL acima de 0,30).

Após testagens de dimensionalidade, seguiu-se com a aplicação da solução fatorial para buscar evidências do modelo teórico. A primeira testagem foi realizada com a configuração para quatro domínios, conforme a versão original do instrumento.

A Tabela 3 apresenta as cargas fatoriais e comunalidade do modelo com quatro domínios. Adotou-se como carga fatorial substancial valores iguais ou maiores de 0,40 para os itens. A comunalidade aceitável é próxima de 0,40^(13)^.

**Tabela 3-:** Cargas fatoriais e comunalidades em modelo com quatro domínios. São Paulo, SP, Brasil, 2023

Variáveis	Λ* Fator 1	Λ* Fator 2	Λ* Fator 3	Λ* Fator 4	h2 ^†^
V ^‡^ 1	**0,91**	-0,17	0,02	0,03	**0,69**
V ^‡^ 2	**0,94**	0,02	-0,12	-0,10	**0,72**
V ^‡^ 3	**0,95**	-0,09	0,03	-0,18	**0,73**
V ^‡^ 4	**0,63**	-0,08	0,18	0,27	**0,71**
V ^‡^ 5	**0,88**	-0,02	-0,02	0,04	**0,76**
V ^‡^ 6	**0,81**	0,06	0,04	-0,09	**0,70**
V ^‡^ 7	**0,90**	0,15	-0,07	-0,14	**0,84**
V ^‡^ 8	**0,80**	0,12	-0,04	0,04	**0,77**
V ^‡^ 9	**0,78**	0,08	0,02	0,01	**0,72**
V ^‡^ 10	**0,57**	0,13	0,22	0,09	**0,71**
V ^‡^ 11	**0,60**	0,07	0,06	**0,32**	**0,74**
V ^‡^ 12	**0,68**	-0,02	0,13	0,20	**0,72**
V ^‡^ 13	**0,40**	0,13	0,05	**0,43**	**0,64**
V ^‡^ 14	**0,33**	**0,40**	-0,12	**0,33**	**0,58**
V ^‡^ 15	**0,72**	0,16	0,01	0,18	**0,85**
V ^‡^ 16	**0,67**	0,07	0,14	0,11	**0,73**
V ^‡^ 17	**0,58**	0,15	0,12	0,15	**0,71**
V ^‡^ 18	**0,70**	0,01	0,14	0,06	**0,67**
V ^‡^ 19	**0,75**	0,09	0,01	0,08	**0,73**
V ^‡^ 20	**0,76**	0,05	0,13	0,03	**0,80**
V ^‡^ 21	**0,46**	0,07	**0,32**	0,30	**0,80**
V ^‡^ 22	0,27	0,22	0,25	-0,10	0,37
V ^‡^ 23	0,13	**0,45**	**0,38**	-0,12	**0,64**
V ^‡^ 24	0,08	0,04	**0,86**	-0,04	**0,84**
V ^‡^ 25	-0,06	0,01	**0,90**	0,06	**0,78**
V ^‡^ 26	0,23	0,25	**0,54**	-0,15	**0,71**
V ^‡^ 27	0,01	**0,89**	0,06	-0,01	**0,86**
V ^‡^ 28	0,07	**0,62**	0,09	0,04	**0,53**

*Λ = Carga fatorial; ^†^h^2^ = Comunalidade; ^‡^V = Variável

Não há ajuste do modelo com quatro domínios, há concentração de 20 itens no primeiro domínio, sendo que os itens 11, 13, 14, 21 e 23 têm dupla saturação (mede dois fatores, considerada violação técnica). O item 22 não apresenta carga fatorial mínima de 0,30 e a comunalidade abaixo de 0,40. Portanto, não há evidências de configuração ajustada com quatro domínios para a amostra do estudo.

Como o princípio de ajuste exploratório permite e recomenda a testagem de configurações diferentes dos instrumentos, aplicou-se a solução fatorial com a parametrização com apenas uma dimensão. Apresentam-se na Tabela 4 os valores das cargas fatoriais e comunalidades.

Todos os itens apresentaram cargas fatoriais adequadas e com valores acima de 0,50. Os itens 22, 25 e 28 apresentam comunalidade abaixo de 0,40, mas próxima dos cortes. Como as cargas fatoriais desses itens são adequadas, optou-se pela manutenção dos mesmos.

**Tabela 4-:** Cargas fatoriais e comunalidades em modelo unidimensional. São Paulo, SP, Brasil, 2023

**Variáveis**	**Λ** *	**h2** ^†^
V ^‡^ 1	0,78	0,61
V ^‡^ 2	0,79	0,63
V ^‡^ 3	0,80	0,64
V ^‡^ 4	0,80	0,65
V ^‡^ 5	0,84	0,72
V ^‡^ 6	0,81	0,66
V ^‡^ 7	0,87	0,76
V ^‡^ 8	0,86	0,74
V ^‡^ 9	0,84	0,70
V ^‡^ 10	0,84	0,71
V ^‡^ 11	0,82	0,68
V ^‡^ 12	0,83	0,68
V ^‡^ 13	0,72	0,52
V ^‡^ 14	0,68	0,46
V ^‡^ 15	0,90	0,83
V ^‡^ 16	0,85	0,73
V ^‡^ 17	0,84	0,71
V ^‡^ 18	0,81	0,67
V ^‡^ 19	0,84	0,71
V ^‡^ 20	0,89	0,80
V ^‡^ 21	0,86	0,74
V ^‡^ 22	0,56	0,31
V ^‡^ 23	0,68	0,46
V ^‡^ 24	0,68	0,47
V ^‡^ 25	0,61	0,37
V ^‡^ 26	0,72	0,52
V ^‡^ 27	0,70	0,49
V ^‡^ 28	0,61	0,37

*Λ = Carga fatorial; ^†^h^2^ = Comunalidade; ^‡^V = Variável

Índices de ajuste podem ser considerados para avaliar a adequação e a qualidade de uma solução fatorial na AFE. Os resultados apresentados pelo modelo unidimensional foram considerados bons, cujos índices: CFI (0,996; IC95% = 0,994 - 0,998) e NNFI (0,996; IC95% = 0,994 - 0,998) indicaram excelente ajuste do modelo. As medidas RMSEA (RMSEA = 0,040; IC95% = 0,0275 - 0,0460), GFI (0,991, IC 95% = 0,990:0,994) e AGFI (0,991, IC95% = 0,989 - 0,993) foram adequadas.

Os índices de confiabilidade apresentados foram: alfa de Cronbach = 0,97 e ômega de McDonald = 0,97, ambos acima de 0,70, indicaram confiabilidade adequada^(13)^.

## Discussão

Este estudo relatou o processo de tradução e adaptação da AICLS do inglês para o contexto brasileiro. Embora reconheça-se tratar de uma escala nova, identificou-se apenas um estudo japonês que realizou tradução e adaptação cultural da AICLS, de forma a viabilizar a comparação da referida escala^(27)^.

A ATC foi guiada por recomendações de adaptação transcultural para instrumentos de medida^(10)^, amplamente utilizadas nesse tipo de estudo, com aceitabilidade internacional dessa metodologia^(28)^. A ATC exige grande rigor metodológico, sendo imprescindível que os valores refletidos em cada item sejam equivalentes entre uma cultura e outra^(29)^.

Equivalências semânticas e culturais devem ser consideradas no processo de ATC. As divergências encontradas nas versões apresentadas pelos tradutores foram solucionadas na síntese das traduções e posteriormente enviada para a fase de retrotradução, recomendada como indicador de qualidade de evidência psicométrica, e permite identificar possíveis discrepâncias^(28)^, destacar inconsistências ou erros conceituais na tradução^(30)^. A versão conciliada não apresentou interpretações equivocadas dos itens, confirmada pela autora do instrumento original.

Nesse contexto, a qualidade do processo de tradução/adaptação é crucial para garantir a validade e a utilidade do teste adaptado, por meio de processo rigoroso, cujo objetivo é manter a equivalência de conteúdo e significado cultural entre o teste original e o traduzido/adaptado e promover a comparabilidade das pontuações entre participantes de diferentes grupos culturais^(31)^.

A análise das evidências de validade relacionadas ao conteúdo da versão brasileira EALCI foi realizada em etapas. Para a validação de conteúdo, um comitê de especialistas interprofissionais, com *expertise* em liderança e prática interprofissional colaborativa, elucidou as divergências e sugeriu ajustes para melhor compreensão. No delineamento de um instrumento coerente para a população a qual irá se destinar, essa fase é imprescindível^(32)^.

Diferente do instrumento original, no qual as autoras relataram que apenas um item foi revisado quanto à sua redação, a versão brasileira teve 12 itens que necessitaram de revisão e demandou realizar uma segunda rodada com o comitê de especialistas. Após a segunda avaliação, os itens tiveram seus conteúdos validados. Na versão original, as autoras utilizaram o Índice de Validade de Conteúdo (IVC), no entanto, ele pode inflar os resultados a partir da junção das respostas com pontuações de 3 e 4 oriundas da análise dos especialistas^(33)^. Neste estudo, utilizou-se o cálculo do CVR, método mais robusto para estabelecer e quantificar a validade de conteúdo^(14)^ e que adequa a concordância ao número de especialistas, assegurando a qualidade da avaliação^(26)^.

Durante o teste-piloto, seguindo as recomendações da literatura^(10)^, cujo objetivo é verificar se os itens, as instruções e a escala de resposta estavam compreensíveis, sobretudo por aqueles a quem se destina o instrumento^(30)^, identificou-se que o item 7 foi o de menor compreensão entre os respondentes, devido a uma expressão que pode ser considerada comum no meio acadêmico, mas que não teve aderência ao público-alvo e que necessitou ser retirada (*status quo*).

Na validação da estrutura interna, necessária para verificar se os atributos de medida correspondem aos atributos teóricos^(21)^ e para o ajuste do modelo, utilizou-se múltiplas testagens, atendendo às recomendações contemporâneas de evidência de validade^(12)^. Também foi analisada se a estrutura fatorial estava adequadamente representada pela sua dimensionalidade^(21)^.

Na versão brasileira, o modelo de quatro dimensões não foi reproduzido. A divergência encontrada na análise paralela indicou testagens adicionais da dimensionalidade, devido às recomendações na literatura em que a análise da dimensionalidade não deve se limitar a uma técnica^(34)^. A unidimensionalidade permaneceu e na AFE, o modelo unidimensional foi o mais adequado e não indicou a manutenção das dimensões na população deste estudo. Cabe ressaltar que, nas análises deste estudo, seguiu-se padrões robustos descritos na psicometria contemporânea, análises consideradas de excelência no desenvolvimento e adaptações de instrumentos^(11-12)^.

Na análise do instrumento original^(5)^ as autoras indicaram, preliminarmente, que a dimensão Capacidade de liderar provavelmente não se manteria no modelo final, visto que vários itens indicavam avaliar um pequeno aspecto do construto. A amostra do estudo original não obteve o número mínimo indicado na literatura, com apenas 101 profissionais, aspecto que pode ter impactado nos resultados das análises realizadas e que explicam as diferenças entre os dois estudos. Outra diferença é que não realizaram análises de dimensionalidade, testes de AFE e AFC, e optaram por testagem do alfa de Cronbach, considerado, neste estudo, como insuficiente para análise da adequação do modelo original.

Assim, neste estudo, optou-se por técnicas de análises diversas às usadas no modelo original, visto que se reconhece a existência de técnicas mais modernas indicadas na literatura especializada, e que apesar da escolha de seguir em processos de análise distintos do instrumento original, considerou-se a EALCI um instrumento basilar, com contribuições para a realidade e para o contexto brasileiro, dado seu arcabouço teórico, ampla tradição e aplicação na prática de construtos de interprofissionalidade^(2)^.

É importante destacar que na versão em japonês^(27)^ também não se encontrou a mesma dimensionalidade do instrumento original AICLS. Após a análise fatorial exploratória, utilizando os 28 itens, divididos em quatro fatores, o instrumento foi reduzido para dez itens, distribuídos em três dimensões. O instrumento final, denominado AICLS-J, foi aplicado em uma amostra de 675 participantes.

Culturas diferentes podem compreender, vivenciar ou expressar um mesmo construto de maneiras distintas e implicam em agrupamento dos itens em dimensões diferentes daquelas observadas na versão original. Na temática da interprofissionalidade, dois instrumentos não reproduziram a dimensionalidade do instrumento original no contexto brasileiro. Na adaptação do instrumento *Readiness for Interprofessional Learning Scale* (RIPLS), a estrutura original de quatro fatores não foi reproduzida igualmente ao modelo original, e indicou, na análise fatorial, uma versão com 27 itens distribuídos em três dimensões: trabalho em equipe e colaboração, identidade profissional e atenção à saúde centrada no paciente^(35)^. O outro instrumento de tradução e adaptação transcultural, *Interprofessional Collaboration Measurement Scale* (IPC), os três fatores da versão original não se replicaram nas análises fatoriais exploratória e confirmatória na amostra brasileira, e indicou tratar-se de um instrumento unidimensional^(36)^, semelhante ao encontrado neste estudo.

Em ambos os casos, a dificuldade em reproduzir as mesmas dimensões da versão original podem ser atribuídas às diferenças culturais, contextos educacionais e de práticas distintas, visto que se tratam de construtos com múltiplos aspectos interdependentes. Diante do referido pressuposto, isso pode ocorrer não apenas entre países, mas também entre regiões do mesmo país, entre diferentes tipos de serviços da saúde. Há concordância de que diferenças de dimensionalidade não devam ser consideradas como uma limitação, mas possam refletir que, no cenário de aplicação da pesquisa, o construto foi percebido como um construto holístico, ou até mesmo indicar lacunas na formação e na prática interprofissional em saúde^(36)^. Reforça-se, portanto, e corrobora-se as diretrizes da área, segundo as quais deve haver uma análise das equivalências de estrutura interna e da medida da escala, considerando as diferenças regionais^(12)^, e que esta análise deve ser realizada quando se opta por utilizar uma escala de medida.

Assim, infere-se que a liderança colaborativa interprofissional pode ser influenciada por aspectos da estrutura organizacional, relações de poder, formação profissional e cultura institucional que variam significativamente entre países, regiões e tipos de serviços. O processo de adaptação permite que o instrumento seja sensível às especificidades linguísticas, culturais e sociais do seu contexto, mantendo os fundamentos conceituais e teóricos do instrumento original, e contribuindo no avanço da pesquisa e na aplicabilidade de novos modelos de gestão, pautados na organização interprofissional das equipes.

Nas análises de evidências de confiabilidade da versão unidimensional brasileira da EALCI o coeficiente ômega de McDonald e alfa de Cronbach foram superiores a 0,70. A versão japonesa utilizou apenas o alfa de Cronbach, com valor = 0,987^(27)^. No presente estudo, optou-se por demonstrar dois indicadores para aumentar a precisão da interpretação.

Quando comparado o contexto dos itens do instrumento CSLS^(8)^ com a versão brasileira da EALCI, embora a estrutura hierárquica não seja avaliada, percebe-se a aproximação entre os aportes teóricos dos dois instrumentos e entre os itens, referentes à colaboração entre os membros da equipe, com seus papéis bem estabelecidos, incentivando a criatividade e discussões no planejamento de cuidados, assim como compartilhamento de conhecimento, habilidades e *expertise*, de acordo com as necessidades de cada situação.

Assim, o instrumento traduzido e validado para o português corrobora na direção de que a liderança colaborativa é fundamental, e prepara líderes de equipes interprofissionais para coordenar e colaborar nas equipes, respeitando o paciente e família acerca das próprias decisões de cuidado, como o caminho para ofertar assistência à saúde de qualidade e com segurança. Trabalhar de forma interprofissional e colaborativa requer a corresponsabilização de todos pelos resultados produzidos^(5)^.

Ter disponível na literatura nacional e internacional um instrumento capaz de medir, de forma adequada, o construto da liderança colaborativa interprofissional é um avanço no campo da interprofissionalidade. O mesmo pode inclusive ser utilizado em programas de treinamento e formação profissional, que se propõem a abordar o desenvolvimento dessa competência, sobretudo na perspectiva do envolvimento dos pacientes e cuidadores nos processos de tomada de decisão, na perspectiva de melhorar a experiência do paciente, a qualidade e segurança do cuidado, prática que ainda não se encontra plenamente consolidada nos serviços de saúde^(37)^.

Como limitações do estudo, cita-se que existem poucas análises do instrumento traduzido para outros idiomas e que a própria versão canadense teve uma amostra aquém do indicado na literatura. Identificou-se apenas o estudo japonês^(27)^, e este estudo tenta avançar para suprir a lacuna identificada, com indicações futuras de novas aplicações e análises da EALCI. Desconhecem-se outros instrumentos que mensuram a liderança colaborativa interprofissional ou outras escalas semelhantes validadas no Brasil, o que não permite comparações.

Ainda que a dimensionalidade da escala original não tenha sido replicada, aspecto que pode ser considerado uma limitação, reforça-se que se seguiu testes de análises mais contemporâneos e diferentes dos aplicados, por considerá-los mais pertinentes no presente estudo para este tipo de escala. Destaca-se que dentre as responsabilidades dos pesquisadores está em apresentar os resultados com respeito à transparência e aos princípios éticos que regem a prática científica, mesmo que não tenham sido consonantes ao dos estudos originais. Esses resultados também contribuem para o avanço do conhecimento e no aprimoramento da escala que mensura a liderança colaborativa interprofissional. Assim, as limitações encontradas devem ser consideradas nas futuras pesquisas e no aprimoramento da EALCI.

Outra limitação é de que as interações entre os profissionais que compunham as equipes nas áreas de atuação no estudo não foram analisadas, aspecto que poderia configurar uma análise da validade de critério. Dessa maneira, há indicações de novas pesquisas, tanto para a aplicação do instrumento traduzido e validado em outros contextos de prática interprofissional bem como no que diz respeito à validade convergente^(38)^, para estabelecer correlação do instrumento com instrumentos que avaliam construtos similares, sobretudo com escalas já validadas e amplamente utilizadas de prática colaborativa interprofissional.

Como implicações para a prática, indica-se que a EALCI possa ser utilizada no contexto brasileiro para a medida global da liderança colaborativa interprofissional e nos procedimentos de análise contemporâneos nos diferentes contextos de cuidados em saúde, para fortalecer a sua aplicabilidade.

## Conclusão

A EALCI, composta por 28 itens, demonstrou ser um instrumento para medir a liderança colaborativa interprofissional, com estrutura unidimensional e apresentou evidências adequadas de validade de conteúdo, processo de resposta, estrutura interna e confiabilidade. É uma escala válida para fazer diagnósticos situacionais, apoiar políticas institucionais de intervenção e de impacto dos programas educacionais na perspectiva da prática interprofissional.

A liderança colaborativa é competência indispensável no trabalho em equipe interprofissional, considerada essencial no cuidado em saúde para a garantia da centralidade do paciente e família. A disponibilização desta escala contribuirá com a compreensão dos processos envolvidos nessa forma de liderança, sua ocorrência nas equipes interprofissionais, e pode auxiliar as instituições de saúde nos programas de desenvolvimento profissional e contribuir na implementação desse modelo assistencial de cuidado.

## Data Availability

Os conjuntos de dados relacionados a este artigo estarão disponíveis mediante solicitação ao autor correspondente.

# Translation: Evidencias de validez de la versión brasileña de la *Assessment Interprofessional Collaborative Leadership Scale* *

**DOI:** 10.1590/1518-8345.7894.4718

**Authors:** Natalia Dalforno da Silva, Geisa Colebrusco de Souza Gonçalves, Jaqueline Alcantara Marcelino da Silva, Flávio Rebustini, Alexandre Pazetto Balsanelli

**Affiliations:** 1 Universidade Federal de São Paulo, Escola Paulista de Enfermagem, São Paulo, SP, Brasil.; 2 Hospital Sírio Libanês, São Paulo, SP, Brasil.; 3 Universidade Federal de São Carlos, Departamento de Enfermagem, São Carlos, SP, Brasil.; 4 Universidade de São Paulo, Escola de Ciências, Humanidades e Artes, São Paulo, SP, Brasil.

**Keywords:** Liderazgo, Grupo de Atención al Paciente, Relaciones Interprofesionales, Estudio de Validación, Personal de Salud, Gestión de Recursos de Personal em Salud

## Abstract

buscar evidencias de validez de la versión en portugués de Brasil de la *Assessment Interprofessional Collaborative Leadership Scale* y analizar evidencias de contenido, proceso de respuesta y estructura interna.

estudio de evaluación psicométrica que incluyó traducción, retrotraducción y evaluación por un comité de especialistas acerca de la equivalencia lingüística y de contenido. Se realizó una prueba piloto con 40 participantes y, posteriormente, la versión final se aplicó a 318 trabajadores de la salud. Se efectuó un análisis factorial exploratorio y una prueba de confiabilidad mediante el alfa de Cronbach y el omega de McDonald.

en la validación de contenido, cuatro ítems presentaron puntuaciones por debajo del valor crítico. La mayoría de los participantes eran profesionales de enfermería (81%) y fisioterapeutas (10%), y del sexo femenino (79%). La factorabilidad obtenida fue Kaiser-Meyer-Olkin = 0,94 y Esfericidad de Bartlett = 2991,4 con p < 0,05. El análisis paralelo indicó un modelo unidimensional con una varianza explicada del 63,71%. Los índices de ajuste del modelo mostraron niveles satisfactorios y los índices de confiabilidad fueron adecuados (alfa de Cronbach = 0,97 y omega de McDonald = 0,97).

la Escala de Evaluación del Liderazgo Colaborativo Interprofesional, con estructura unidimensional y 28 ítems, presentó buenas evidencias de validez de contenido, proceso de respuesta y estructura interna, y se considera adecuada para medir el liderazgo colaborativo interprofesional.

## Destacados

**(1)** Instrumento para medir el liderazgo colaborativo interprofesional. **(2)** Instrumento para implementar un modelo asistencial de cuidado interprofesional. **(3)** Diagnóstico para fundamentar programas de desarrollo interprofesional. **(4)** Evidencias de validez de un instrumento de liderazgo colaborativo interprofesional.

## Introducción

La práctica interprofesional colaborativa es una forma de organización del trabajo imprescindible para abordar cuestiones de salud complejas y proporcionar atención integral^(1)^. Requiere esfuerzos coordinados para garantizar el intercambio de información y la toma de decisiones de modo que la pericia de cada miembro del equipo se materialice en la implementación de los cuidados y en la atención de las necesidades de salud^(2)^.

Entre las competencias de la práctica interprofesional colaborativa se encuentra el liderazgo colaborativo, en el cual los profesionales trabajan juntamente con los pacientes y las familias para promover la toma de decisiones compartidas y generar corresponsabilidades dentro del equipo^(2)^.

A diferencia del liderazgo tradicional, jerárquico y centralizado, el liderazgo colaborativo interprofesional se basa en el reparto de responsabilidades y en la construcción colectiva y articulada de soluciones a partir de múltiples perspectivas de profesionales y pacientes en las decisiones clínicas, con el fin de mejorar la calidad de la atención^(3)^. También descrito en la literatura como liderazgo colectivo o distributivo, se considera una estrategia para implementar intervenciones complejas, promover una comunicación y participación seguras e involucrar a las partes interesadas en un entorno que busca la equidad^(4)^. No obstante, su aplicación práctica en el día a día de los servicios sigue siendo poco explorada.

En este contexto, el instrumento *Assessment Interprofessional Collaborative Leadership Scale* (AICLS)^(5)^ fue desarrollado y publicado en el ámbito canadiense en 2019 con el propósito de medir el liderazgo colaborativo interprofesional en las instituciones de salud, mediante las percepciones individuales de los profesionales de salud que integran los equipos^(5)^. La génesis de su construcción es el modelo teórico que explora la interrelación entre estructuras verticales y formales de la institución y equipos colaborativos como nuevas posibilidades del ejercicio del liderazgo^(6)^.

Aunque los contextos de formación y práctica en salud de Brasil y Canadá presentan especificidades locales, la literatura sobre trabajo en equipo interprofesional ha establecido ampliamente las competencias interprofesionales necesarias para que este se materialice, tales como comunicación, colaboración y liderazgo colaborativo, en diversos escenarios de salud^(7)^.

Los estudios específicos sobre liderazgo colaborativo interprofesional son escasos y se identifica una laguna en los instrumentos de medición. Se reconoce la existencia de la *Clinical Shared Leadership Scale* (CSLS)^(8)^; sin embargo, esta no evalúa la toma de decisiones compartida con la participación directa de pacientes y cuidadores, característica fundamental identificada en esta competencia, como se observó en una investigación con participación significativa de jóvenes en la propuesta y desarrollo de cuidados integrados basados en liderazgo colaborativo interprofesional^(9)^. Así, el liderazgo colaborativo interprofesional requiere la participación de pacientes y familiares (usuarios del servicio), razón por la cual se optó por la traducción de la AICLS^(5)^, dada su pertinencia y relevancia teórica para la realidad y el contexto del sistema de salud brasileño.

El instrumento AICLS, en Canadá, fue sometido a análisis de validez de contenido y se consideró altamente relevante en su versión preliminar; su evidencia de confiabilidad fue de 0,96 y sus dimensiones variaron de 0,85 a 0,92 [Relación simbiótica = 0,87; Atención plena (*Mindfulness*) = 0,92; Recursos compartidos = 0,92 y Capacidad de liderar = 0,85]^(5)^.

Por lo tanto, resulta relevante la traducción y validación de este instrumento con el supuesto de que será una medida eficaz para analizar el liderazgo colaborativo en equipos interprofesionales y que puede incidir en la comprensión de esta competencia para lograr la mejora de la calidad y la seguridad de la atención proporcionada por los equipos. En este sentido, el objetivo de este estudio fue buscar evidencias de validez de la *Assessment Interprofessional Collaborative Leadership Scale* (AICLS) para el portugués de Brasil y analizar evidencias de contenido, proceso de respuesta y estructura interna.

## Método

### Tipo del estudio

Se trata de un estudio de evaluación psicométrica, de evidencias de validez y adaptación transcultural (ATC)^(10)^ mediante las fases: traducción; síntesis, retrotraducción, evaluación por comité de especialistas, prueba piloto del instrumento y, por último, análisis de las evidencias de validez del instrumento^(10-11)^. El estudio siguió las directrices contempladas en los *Test Adaptation Reporting Standards* (TARES)^(12)^.

La traducción fue realizada por dos traductores independientes, con la elaboración de una versión síntesis única a cargo de un tercer traductor^(10)^, evaluada y aprobada por la autora del instrumento.

La equivalencia de la versión en portugués se estableció a partir de cuatro dimensiones: semántica, idiomática, cultural y conceptual, por 17 especialistas en práctica interprofesional colaborativa y/o traducciones de instrumentos de medida. Tras la aprobación de este comité de especialistas, se procedió a la prueba piloto con 40 trabajadores asistenciales o de gestión que componían equipos de unidades de internación de un hospital.

Para la evaluación de las propiedades psicométricas, uso y adopción del instrumento AICLS^(5)^ en Brasil, se buscó reunir evidencias de validez basadas en el contenido, proceso de respuesta, estructura interna y verificación de la confiabilidad.

La validación de contenido comprende la evaluación cualitativa (comité de especialistas) y la evaluación cuantitativa de cada ítem, considerando la importancia individual del ítem en la composición del instrumento y la representación del constructo^(13)^, mediante el cálculo del *Content Validity Ratio* (CVR)^(14)^.

En la validación del proceso de respuesta se verifica cómo se interpreta el ítem y su relación con el constructo medido^(11)^. Los participantes de la prueba piloto analizaron la viabilidad, comprensibilidad y facilidad de uso del instrumento. Se registró el tiempo de respuesta, dato que puede aportar evidencias sobre la complejidad de los procesos de respuesta^(11,15-16)^.

El análisis de la estructura interna del constructo se realizó mediante Análisis Factorial Exploratorio (AFE), que evalúa, mediante las cargas factoriales, la pertinencia de cada ítem para el factor^(16)^. La retención de factores influye sustancialmente en los resultados del AFE y tiene fuertes implicaciones teóricas, pues predetermina la dimensionalidad asumida de un constructo latente. Debido a su naturaleza exploratoria, el AFE se utiliza en casos cuya comprensión de la estructura factorial del concepto latente aún es incipiente^(17)^, como es el caso de la AICLS, recién descrita en la literatura^(5)^. Se emplearon índices de ajuste de la solución factorial para apoyar la determinación del número de factores retenidos^(17)^ y la adecuación de la estructura factorial encontrada^(18)^.

La confiabilidad también fue verificada utilizando dos indicadores, según la recomendación de la literatura, para aumentar la confiabilidad de la interpretación^(19)^.

### Lugar

El estudio se llevó a cabo en un hospital general privado, de nivel terciario y de gran porte, situado en el municipio de São Paulo (SP), Brasil, considerado un centro de referencia internacional en salud.

### Población y muestra del estudio

La población del estudio estuvo compuesta por trabajadores de la salud, en cargos gerenciales y asistenciales, que conformaban equipos interprofesionales de unidades críticas y de internación, clínicas y quirúrgicas.

El tamaño de la muestra se calculó con base en las recomendaciones para la verificación de la estructura interna de instrumentos, que indican un mínimo de 300 participantes^(13)^. En este estudio se alcanzó una muestra por conveniencia de 318 trabajadores de la salud que integraban los equipos: enfermeros, técnicos de enfermería, fisioterapeutas, médicos, farmacéuticos, psicólogos y nutricionistas.

El criterio de inclusión fue trabajadores con al menos un año de desempeño en la institución, en cargos de gestión o asistencia, con actividades compartidas con al menos un trabajador de otra área de formación. El criterio de exclusión fue trabajadores que estaban de licencia en el momento de la recolección de datos.

### Variables del estudio

El AICLS consta de 28 ítems, divididos en cuatro dimensiones: Relación simbiótica; Atención plena (*Mindfulness*); Recursos compartidos; y Capacidad de liderar^(5)^.

La dimensión Relación simbiótica, compuesta por cinco ítems, se define como una colaboración en la que todos los miembros del equipo tienen sus funciones bien establecidas y se adaptan recíprocamente a las demandas que surgen^(5)^.

La dimensión Atención plena (*Mindfulness*) está formada por nueve ítems y se caracteriza por un enfoque atento y prolongado, con atención a las situaciones y experiencias inmediatas a medida que aparecen^(5)^.

La dimensión Recursos compartidos está compuesta por siete ítems y se define por un entorno que incentiva la apertura entre los profesionales para compartir conocimientos, habilidades y experiencia dentro de un equipo^(5)^.

La dimensión Capacidad de liderar, compuesta por siete ítems, se define como la disposición a liderar y aceptar la responsabilidad inherente al cargo de liderazgo^(5)^.

Los encuestados indican su nivel general de acuerdo con la ocurrencia de cada ítem en una escala Likert de cinco puntos: 1 – Nunca; 2 – Raramente; 3 – Ocasionalmente; 4 – La mayoría de las veces; y 5 – Siempre. Las clasificaciones producen puntuaciones de 28 a 140^(5)^.

### Recolección de datos

La versión elaborada tras las etapas de validación de contenido y proceso de respuesta se transformó en un cuestionario electrónico, junto con preguntas de caracterización de la muestra. Los datos se recolectaron mediante la plataforma *Research Electronic Data Capture* (RedCap)^(20)^. La invitación para participar se envió por correo electrónico a las coordinadoras de área, quienes la remitieron a los profesionales/trabajadores de la salud de sus respectivos equipos.

### Período

El periodo de recolección de datos transcurrió entre el 15 de julio y el 15 de septiembre de 2023.

### Tratamiento y análisis de los datos

Para iniciar el AFE y verificar la factorabilidad de los datos, se calculó el índice Kaiser-Meyer-Olkin (KMO) y se realizó el Test de Esfericidad de Bartlett (TEB)^(13)^.

Para la extracción de factores se empleó *Unweighted Least Squares* (ULS) con rotación *oblimin*, a partir de una matriz de correlación policórica^(13)^; posteriormente se exploró la dimensionalidad del instrumento mediante la técnica de análisis paralelo, *Optimal Implementation of Parallel Analysis* (PA), enfoque recomendado para un conjunto de variables ordinales con puntuación politómica^(21)^, el método de Hull con *Comparative Fit Index* ^(22)^ y la aplicación de *Closeness of dimensionality* ^(23)^, compuesta por tres indicadores: *Item Unidimensional Congruence* (I-UniCo), *Item Explained Common Variance* (I-ECV) y *Mean of Item Residual Absolute Loadings* (MIREAL). Valores de I-UniCo > 0,95, I-ECV > 0,85 y MIREAL < 0,30 indican que los datos pueden tratarse como esencialmente unidimensionales^(23)^.

La solución factorial se aplicó mediante modelado de ecuaciones estructurales y, para comprobar la necesidad de ajustes, se calcularon los índices *Root Mean Square Error of Approximation* (RMSEA), *Non-Normed Fit Index* (NNFI), *Comparative Fit Index* (CFI), *Goodness of Fit Index* (GFI) y *Adjusted Goodness of Fit Index without diagonal values* (AGFI)^(24)^. La confiabilidad se verificó mediante los índices alfa de Cronbach y omega de McDonald^(19)^.

Todos los análisis se realizaron con el *software Factor*.

### Aspectos éticos

Los aspectos éticos de la investigación se cumplieron según la Resolución n.º 466 de 2012^(25)^, tanto en la fase de evaluación del comité de especialistas como en la aplicación de la prueba piloto y el pretest. Dichas etapas se llevaron a cabo tras la aprobación del Comité de Ética en Investigación (CEI) de la *Universidade Federal de São Paulo* y de la institución coparticipante, el hospital donde se realizó el estudio, conforme a los informes n.º 5.533.152 y n.º 5.571.337, respectivamente.

## Resultados

### Adaptación transcultural y validación de contenido

El análisis de equivalencia mostró que el 62,5% de los 28 ítems presentaron concordancia superior al 80%. Tras la primera ronda de análisis del comité de especialistas, los 12 ítems cuya tasa de concordancia fue inferior a 0,80 fueron revisados, así como el nombre del instrumento, las dos primeras frases explicativas de la introducción y el párrafo final.

Se acogieron las sugerencias y, después de las modificaciones, se realizó una nueva ronda con el comité de especialistas para revisar dichos ítems y elegir, entre dos opciones, la mejor alternativa, considerando sus conceptos y significados dentro de la cultura brasileña. La definición final de los 12 ítems se describe en la Figura 1.

**Figura 1-:** Modificaciones realizadas por los autores en los ítems tras las sugerencias y la segunda ronda del comité de especialistas. São Paulo, SP, Brasil, 2022

Ítem tras la modificación	**Modificación**
Sección 1: RELACIÓN SIMBIÓTICA: La relación simbiótica es una colaboración en la que todos los miembros del equipo tienen sus propios roles bien establecidos y se adaptan mutuamente a la dinámica cambiante de las demandas.	Se cambió la palabra “ *ambos* ” por “ *todos* ” y se alteró la posición de la palabra “ *dinâmica* ”.
2. Incentiva a los miembros del equipo a valorar la *expertise* de los demás.	Se cambió la palabra “ *compet* ê *ncias* ” por “ *expertise* ”.
3. Incentiva a los miembros del equipo a acercar sus capacidades complementarias (compartiendo conocimientos, habilidades y experiencias) para orientar el plan de cuidados.	Se cambió la palabra “ *explorar* ” por “ *aproximar* ”, se invirtió el orden de las palabras “ *compartilhando conocimientos* ” y se cambió “ *abordar o planejamento* ” por “ *direcionar o plano* ”.
Sección 2: ATENCIÓN PLENA ( *Mindfulness* ): La Atención Plena es el enfoque consciente y prolongado de la atención en experiencias inmediatas a medida que ocurren.	Se añadió “ *Mindfulness* ” y se cambió la palabra “ *intencional* ” por “ *consciente* ”.
7. Incentiva a los miembros del equipo a centrarse más allá del status quo (es decir, la forma habitual de hacer las cosas) en cuestiones relevantes y esenciales del cuidado.	Se cambió “ *essência* ” por “ *esenciais* ”.
8. Incentiva a los miembros a considerar soluciones creativas para la planificación de cuidados complejos a pacientes/clientes.	Se cambió “ *atendimentos mais* ” por “ *cuidados* ”.
11. Es receptivo para apoyar los cambios sugeridos por los miembros del equipo.	Se invirtió el orden de la palabra “ *apoio* ”.
16. Incentiva a los miembros del equipo a establecer metas compartidas para el trabajo en equipo.	Se cambió “ *sobre seu* ” por “ *para o* ”.
21. Comparte el trabajo entre los miembros del equipo, de acuerdo con sus capacidades, cuando se implementan los planes de cuidado	Se invirtió el orden de las palabras al inicio de la frase.
22. Los miembros del equipo apoyan que los pacientes/clientes sean líderes colaborativos	Se cambió “ *apoiem* ” por “ *apoiam* ”.
24. Todos los miembros del equipo aceptan y se responsabilizan de su trabajo compartido en equipo	Se cambió “ *responsabilidade* ” por “ *se responsabilizam* ”.
27. Hay apoyo para la rotación del líder del equipo de acuerdo con las necesidades de nuestra planificación de cuidados	Se cambió “ *apoiemos* ” por “ *h* á *apoio* ” y “ *conforme* ” por “ *de acordo* ”.

En la validación de contenido cuantitativa, el valor crítico del CVR aceptable para 17 especialistas fue de 0,529^(26)^, lo que indicó la adherencia del ítem en este estudio. Cuatro ítems obtuvieron puntuaciones por debajo de 0,529 en alguna de las categorías de evaluación: relevancia teórica, pertinencia práctica, suficiencia de cobertura y comprensibilidad en el conjunto y la dimensionalidad.

El ítem 2 de la sección 1, Incentiva a los miembros del equipo a centrarse más allá del *status quo* (es decir, la forma habitual de hacer las cosas) en cuestiones relevantes y esenciales del cuidado, presentó puntuaciones inferiores a 0,529 en relevancia teórica, pertinencia práctica y cobertura.

El ítem 19 de la sección 3, El proceso de toma de decisiones se centra en objetivos compartidos por todos los miembros del equipo, obtuvo una puntuación menor en la evaluación de cobertura.

En la sección 4, dos ítems registraron puntuaciones inferiores a 0,529. El ítem 22, Los miembros del equipo apoyan que los pacientes/clientes sean líderes colaborativos, recibió puntuaciones menores en pertinencia práctica, cobertura y dimensionalidad; y el ítem 23, Los miembros del equipo están dispuestos a asumir la función de liderazgo del equipo cuando se les solicita, en cobertura y dimensionalidad. Todos los ítems se mantuvieron en el instrumento en esta etapa, dado que presentaron valores por debajo de la razón considerada solo en alguna de las categorías evaluadas y no en su totalidad.

### Validación del proceso de respuesta

La validez del proceso de respuesta, realizada mediante entrevista en la prueba piloto, certificó la viabilidad del instrumento para su uso. Los participantes emplearon, en promedio, nueve minutos y 27 segundos en contestar el instrumento. La desviación estándar fue de tres minutos y quince segundos y el tiempo máximo de respuesta fue de 15 minutos y 51 segundos.

Los encuestados presentaron dificultad con el término “*status quo”* en el ítem 7, en la sección 2, a pesar de que la explicación figuraba entre paréntesis dentro del mismo ítem, conforme lo indicó el comité de especialistas. Como sugerencia, se retiró el término”*status quo*” y ese ítem quedó definido como Incentiva a los miembros del equipo a desarrollar procesos que conduzcan a la creación de un entorno donde las decisiones sean compartidas.

La comprensión de las opciones de respuesta de los ítems fue adecuada para el 100% de los participantes, así como la organización general de los ítems. En la evaluación de la cobertura de los ítems, el 87,5% de los participantes la consideró adecuada y uno de ellos sugirió la eliminación de ítems que juzgaba similares.

En cuanto a la dificultad de completar el instrumento, el 62,5% de los participantes informó no haber encontrado dificultades y se registraron 20 sugerencias que fueron consideradas. De este modo, se ajustaron ocho ítems (ítems 1 y 3 de la sección 1; ítems 7, 9 y 10 de la sección 2; ítems 19 y 21 de la sección 3; e ítem 25 de la sección 4).

La versión final del instrumento, tras las modificaciones sugeridas, fue respondida por 318 participantes; seis señalaron tener menos de un año en la institución, lo que configuraría su exclusión tras la recopilación de datos, sin embargo, debido al pequeño porcentaje (1,8%) respecto al total de la muestra, se optó por mantenerlos en la presentación de los resultados.

En cuanto a las características sociodemográficas, cabe destacar que la muestra estuvo compuesta mayoritariamente por personas del sexo femenino (79%) y en el rango etario de 31-40 años (44%). La carga horaria laboral semanal de 30-40 horas fue prevalente (67%). Respecto al mayor nivel de titulación, el 53% de los participantes tenía especialización. Los enfermeros (44%), seguidos de los técnicos de enfermería (37%) y fisioterapeutas (10%), fueron los profesionales con mayor participación. En relación con el tiempo de ejercicio profesional, el intervalo de 11-15 años fue predominante (24%) y el tiempo de actuación en la unidad fue de uno a tres años (31% de los participantes). En cuanto al tipo de unidad de actuación, los participantes trabajaban en la Unidad Semiintensiva (26%), seguida de la Unidad de Cuidados Intensivos (21%).

### Validación de la estructura interna

Se procedió al Análisis Factorial Exploratorio (AFE) con base en la matriz policórica y se verificó si los ítems eran factorizables mediante la *Measure of Sample Adequacy* (MSA). En la modelización inicial utilizando los 28 ítems del instrumento, se hallaron buenos índices de adecuación: TEB = 2991,4 (gl = 378; P = 0,000010) y KMO = 0,94977 con *bootstrap* intervalo de confianza del 95% para el KMO = (0,573 - 1,204).

El primer estudio de la dimensionalidad/factores del instrumento se realizó mediante el análisis paralelo (AP), que indicó la existencia de una sola dimensión, con una varianza explicada del 63,71% de la variable latente. Debido a la divergencia entre el modelo original y el obtenido por AP, se optó por pruebas adicionales de dimensionalidad.

La segunda técnica empleada fue Hull con el *Comparative Fit Index* (CFI), presentada en la Tabla 1.

Nuevamente, el resultado indicó solamente una dimensión. La tercera prueba adoptada fue la técnica de unidimensionalidad/multidimensionalidad, con los índices UniCo, ECV y REAL presentados a continuación en la Tabla 2.

**Tabla 1-:** Dimensionalidad por Hull con *Comparative Fit Index*. São Paulo, SP, Brasil, 2023

**Número factores**	**CFI** *	**Grados de libertad**	***Scree test***
0	0,00	378	0,00
1	0,99	350	**250,61** *
2	1,00	323	0,00
3	1,00	297	

*CFI = *Comparative Fit Index*

**Tabla 2-:** *Closeness of dimensionality*. São Paulo, SP, Brasil, 2023

**Variables**		**I-UniCo** *	**I-ECV** ^†^	**I-REAL** ^‡^
V ^§^ 1. Ayuda a los miembros a valorar sus contribuciones al trabajo en equipo.		0,99	0,93	0,22
V ^§^ 2. Incentiva a los miembros del equipo a valorar la expertise de los demás.		0,99	0,92	0,23
V ^§^ 3. Incentiva a los miembros del equipo a acercar sus capacidades complementarias (conocimientos, habilidades y experiencias compartidas) para orientar el plan de cuidados.		1,00	0,97	0,14
V ^§^ 4. Permite que todos los miembros del equipo tengan la oportunidad de expresar sus opiniones.		1,00	0,98	0,11
V ^§^ 5. Posibilita que los miembros del equipo vean sus resultados compartidos como significativos y valiosos.		0,99	0,95	0,20
V ^§^ 6. Incentiva a los miembros del equipo a desarrollar procesos que lleven a la creación de un entorno donde las decisiones sean compartidas.		1,00	0,99	0,08
V ^§^ 7. Incentiva a los miembros del equipo a enfocarse más allá de la forma habitual de hacer las cosas, en cuestiones relevantes y esenciales del cuidado.		1,00	0,98	0,13
V ^§^ 8. Incentiva a los miembros a considerar soluciones creativas para la planificación de cuidados complejos a pacientes/clientes.		0,99	0,97	0,16
V ^§^ 9. Incentiva a los miembros del equipo a reevaluar las maneras tradicionales de abordar situaciones similares.		1,00	0,98	0,11
V ^§^ 10. Incentiva discusiones abiertas entre los miembros del equipo sobre cuestiones de la planificación del cuidado.		1,00	0,99	0,04
V ^§^ 11. Es receptivo para apoyar los cambios sugeridos por los miembros del equipo.		0,99	0,97	0,15
V ^§^ 12. Incentiva a los miembros del equipo a adaptarse a diversas situaciones.		1,00	0,98	0,11
V ^§^ 13. Incentiva a los miembros del equipo a cuestionar algo que no tenga sentido.		1,00	0,97	0,12
V ^§^ 14. Apoya la innovación creativa de los miembros del equipo en situaciones de incertidumbre en la planificación del cuidado del paciente/cliente.		1,00	0,98	0,10
V ^§^ 15. Se asegura de que todos los miembros del equipo tengan la oportunidad de compartir sus perspectivas sobre la planificación del cuidado del paciente/cliente.		1,00	0,98	0,13
V ^§^ 16. Incentiva a los miembros del equipo a establecer metas compartidas para el trabajo en equipo.		1,00	0,99	0,05
V ^§^ 17. Facilita el ajuste de las funciones de los miembros del equipo a las necesidades situacionales.		1,00	0,99	0,03
V ^§^ 18. Incentiva a los miembros del equipo a asumir la responsabilidad de sus aportes en el proceso de toma de decisiones del equipo.		1,00	0,99	0,06
V ^§^ 19. Se centra en objetivos compartidos por todos los miembros del equipo en el proceso de toma de decisiones.		1,00	0,97	0,14
V ^§^ 20. Incentiva la integración de perspectivas para facilitar procesos compartidos de toma de decisiones en el desarrollo del plan de cuidado del paciente/cliente.		1,00	0,99	0,06
V ^§^ 21. Comparte el trabajo entre los miembros del equipo, de acuerdo con sus capacidades, cuando se implementan los planes de cuidado.		1,00	0,99	0,05
V ^§^ 22. Los miembros del equipo apoyan que los pacientes/clientes sean líderes colaborativos.		0,98	0,83	0,26
V ^§^ 23. Los miembros del equipo están dispuestos a asumir la función de liderazgo del equipo cuando se les solicita.		0,93	0,72	0,43
V ^§^ 24. Todos los miembros del equipo aceptan y se responsabilizan de su trabajo compartido en equipo.		0,86	0,62	0,54
V ^§^ 25. Todos los miembros del equipo contribuyen a objetivos comunes y compartidos.		0,76	0,54	0,58
V ^§^ 26. Los miembros del equipo se orientan mutuamente para que todos sean capaces de liderar al equipo de forma eficaz.		0,94	0,74	0,43
V ^§^ 27. Hay apoyo para la rotación del líder del equipo, de acuerdo con las necesidades de nuestra planificación de cuidado.		0,98	0,85	0,30
V ^§^ 28. Elegimos juntos al líder de nuestro equipo.		0,99	0,88	0,23
Evaluación	General			
UniCo ^||^	=	0,98		
ECV ^¶^	=	0,92		
MIREAL ^**^	=	0,19		

*I-UniCo = *Item Unidimensional Congruence*; ^†^I-ECV - Item *Explained Common Variance*; ^‡^I-REAL = *Item Residual Absolute Loadings*; ^§^V = Variable; ^||^UniCo = *Unidimensional Congruence*; ^¶^ECV = *Explained Common Variance*; **MIREAL = *Mean of Item Residual Absolute Loadings*

A partir del resultado presentado, el modelo general puede considerarse, nuevamente, unidimensional, cuyos valores de los índices de los ítems 23, 24, 25 y 26 no sustentan la unidimensionalidad (I-REAL por encima de 0,30).

Tras las pruebas de dimensionalidad, se procedió a la aplicación de la solución factorial para buscar evidencias del modelo teórico. La primera prueba se realizó con la configuración de cuatro dominios, conforme a la versión original del instrumento.

La Tabla 3 presenta las cargas factoriales y la comunalidad del modelo con cuatro dominios. Se adoptó como carga factorial sustancial valores iguales o mayores que 0,40 para los ítems. La comunalidad aceptable es cercana a 0,40^(13)^.

**Tabla 3-:** Cargas factoriales y comunalidades en el modelo con cuatro dominios. São Paulo, SP, Brasil, 2023

Variables	Λ* Factor 1	Λ* Factor 2	Λ* Factor 3	Λ* Factor 4	h2 ^†^
V ^‡^ 1	**0,91**	-0,17	0,02	0,03	**0,69**
V ^‡^ 2	**0,94**	0,02	-0,12	-0,10	**0,72**
V ^‡^ 3	**0,95**	-0,09	0,03	-0,18	**0,73**
V ^‡^ 4	**0,63**	-0,08	0,18	0,27	**0,71**
V ^‡^ 5	**0,88**	-0,02	-0,02	0,04	**0,76**
V ^‡^ 6	**0,81**	0,06	0,04	-0,09	**0,70**
V ^‡^ 7	**0,90**	0,15	-0,07	-0,14	**0,84**
V ^‡^ 8	**0,80**	0,12	-0,04	0,04	**0,77**
V ^‡^ 9	**0,78**	0,08	0,02	0,01	**0,72**
V ^‡^ 10	**0,57**	0,13	0,22	0,09	**0,71**
V ^‡^ 11	**0,60**	0,07	0,06	**0,32**	**0,74**
V ^‡^ 12	**0,68**	-0,02	0,13	0,20	**0,72**
V ^‡^ 13	**0,40**	0,13	0,05	**0,43**	**0,64**
V ^‡^ 14	**0,33**	**0,40**	-0,12	**0,33**	**0,58**
V ^‡^ 15	**0,72**	0,16	0,01	0,18	**0,85**
V ^‡^ 16	**0,67**	0,07	0,14	0,11	**0,73**
V ^‡^ 17	**0,58**	0,15	0,12	0,15	**0,71**
V ^‡^ 18	**0,70**	0,01	0,14	0,06	**0,67**
V ^‡^ 19	**0,75**	0,09	0,01	0,08	**0,73**
V ^‡^ 20	**0,76**	0,05	0,13	0,03	**0,80**
V ^‡^ 21	**0,46**	0,07	**0,32**	0,30	**0,80**
V ^‡^ 22	0,27	0,22	0,25	-0,10	0,37
V ^‡^ 23	0,13	**0,45**	**0,38**	-0,12	**0,64**
V ^‡^ 24	0,08	0,04	**0,86**	-0,04	**0,84**
V ^‡^ 25	-0,06	0,01	**0,90**	0,06	**0,78**
V ^‡^ 26	0,23	0,25	**0,54**	-0,15	**0,71**
V ^‡^ 27	0,01	**0,89**	0,06	-0,01	**0,86**
V ^‡^ 28	0,07	**0,62**	0,09	0,04	**0,53**

*Λ = Carga factorial; ^†^h^2^ = Comunalidad; ^‡^V = Variable

No hay ajuste del modelo con cuatro dominios; existe concentración de 20 ítems en el primer dominio, y los ítems 11, 13, 14, 21 y 23 presentan doble saturación (miden dos factores, lo que se considera una violación técnica). El ítem 22 no presenta carga factorial mínima de 0,30 y la comunalidad está por debajo de 0,40. Por lo tanto, no existen evidencias de configuración ajustada con cuatro dominios para la muestra del estudio.

Dado que el principio de ajuste exploratorio permite y recomienda la prueba de configuraciones diferentes de los instrumentos, se aplicó la solución factorial con la parametrización de una sola dimensión. Se presentan en la Tabla 4 los valores de las cargas factoriales y comunalidades.

Todos los ítems presentaron cargas factoriales adecuadas y con valores superiores a 0,50. Los ítems 22, 25 y 28 presentan comunalidad por debajo de 0,40, aunque cercana a los puntos de corte. Como las cargas factoriales de estos ítems son adecuadas, se optó por mantenerlos.

Los índices de ajuste pueden considerarse para evaluar la adecuación y la calidad de una solución factorial en el AFE. Los resultados presentados por el modelo unidimensional se consideraron buenos, cuyos índices CFI (0,996; IC95% = 0,994 - 0,998) y NNFI (0,996; IC95% = 0,994 - 0,998) indicaron un ajuste excelente del modelo. Las medidas RMSEA (RMSEA = 0,040; IC95% = 0,0275 - 0,0460), GFI (0,991; IC95% = 0,990 - 0,994) y AGFI (0,991; IC95% = 0,989 - 0,993) fueron adecuadas.

Los índices de confiabilidad presentados fueron: alfa de Cronbach = 0,97 y ômega de McDonald = 0,97, ambos por encima de 0,70, lo que indicó confiabilidad adecuada^(13)^.

**Tabla 4–:** Cargas factoriales y comunalidades en el modelo unidimensional. São Paulo, SP, Brasil, 2023

**Variable**	**Λ** *	**h2** ^†^
V ^‡^ 1	0,78	0,61
V ^‡^ 2	0,79	0,63
V ^‡^ 3	0,80	0,64
V ^‡^ 4	0,80	0,65
V ^‡^ 5	0,84	0,72
V ^‡^ 6	0,81	0,66
V ^‡^ 7	0,87	0,76
V ^‡^ 8	0,86	0,74
V ^‡^ 9	0,84	0,70
V ^‡^ 10	0,84	0,71
V ^‡^ 11	0,82	0,68
V ^‡^ 12	0,83	0,68
V ^‡^ 13	0,72	0,52
V ^‡^ 14	0,68	0,46
V ^‡^ 15	0,90	0,83
V ^‡^ 16	0,85	0,73
V ^‡^ 17	0,84	0,71
V ^‡^ 18	0,81	0,67
V ^‡^ 19	0,84	0,71
V ^‡^ 20	0,89	0,80
V ^‡^ 21	0,86	0,74
V ^‡^ 22	0,56	0,31
V ^‡^ 23	0,68	0,46
V ^‡^ 24	0,68	0,47
V ^‡^ 25	0,61	0,37
V ^‡^ 26	0,72	0,52
V ^‡^ 27	0,70	0,49
V ^‡^ 28	0,61	0,37

*Λ = Carga factorial; ^†^h^2^ = Comunalidad; ^‡^V = Variable

## Discusión

Este estudio describió el proceso de traducción y adaptación de la AICLS del inglés al contexto brasileño. Aunque se reconoce que se trata de una escala reciente, solo se identificó un estudio japonés que tradujo y adaptó culturalmente la AICLS, lo que posibilita la comparación de dicha escala^(27)^.

La ATC se guio por recomendaciones de adaptación transcultural para instrumentos de medición^(10)^, ampliamente utilizadas en este tipo de estudios y con aceptación internacional de esta metodología^(28)^. La ATC exige un gran rigor metodológico, siendo imprescindible que los valores reflejados en cada ítem sean equivalentes entre una cultura y otra^(29)^.

Las equivalencias semánticas y culturales deben considerarse en el proceso de ATC. Las divergencias encontradas en las versiones presentadas por los traductores se resolvieron en la síntesis de las traducciones y posteriormente se enviaron a la fase de retrotraducción, recomendada como indicador de calidad de evidencia psicométrica, lo que permite identificar posibles discrepancias^(28)^ y destacar inconsistencias o errores conceptuales en la traducción^(30)^. La versión conciliada no presentó interpretaciones equivocadas de los ítems, hecho confirmado por la autora del instrumento original.

En este contexto, la calidad del proceso de traducción/adaptación es crucial para garantizar la validez y la utilidad del test adaptado, mediante un procedimiento riguroso cuyo objetivo es mantener la equivalencia de contenido y significado cultural entre el test original y el traducido/adaptado, y promover la comparabilidad de las puntuaciones entre participantes de distintos grupos culturales^(31)^.

El análisis de las evidencias de validez relacionadas con el contenido de la versión brasileña EALCI se llevó a cabo por etapas. Para la validación de contenido, un comité de especialistas interprofesionales, con experiencia en liderazgo y práctica interprofesional colaborativa, esclareció las divergencias y sugirió ajustes para lograr una mejor comprensión. En la elaboración de un instrumento coherente para la población a la que se destinará, esta fase es indispensable^(32)^.

A diferencia del instrumento original, en el que las autoras informaron que solo un ítem fue revisado en su redacción, la versión brasileña tuvo 12 ítems que requirieron revisión y fue necesario realizar una segunda ronda con el comité de especialistas. Tras esta segunda evaluación, los ítems tuvieron su contenido validado. En la versión original, las autoras utilizaron el Índice de Validez de Contenido (IVC); sin embargo, este puede inflar los resultados al combinar las respuestas con puntuaciones de 3 y 4 provenientes del análisis de especialistas^(33)^. En este estudio se empleó el cálculo del CVR, método más robusto para establecer y cuantificar la validez de contenido^(14)^ y que ajusta la concordancia al número de especialistas, garantizando la calidad de la evaluación^(26)^.

Durante la prueba piloto, que siguió las recomendaciones de la literatura^(10)^ y cuyo objetivo es verificar si los ítems, las instrucciones y la escala de respuesta son comprensibles, sobre todo para quienes se destina el instrumento^(30)^, se identificó que el ítem 7 fue el de menor comprensión entre los encuestados, debido a una expresión que puede considerarse común en el ámbito académico, pero que no tuvo aceptación entre el público objetivo y que fue necesario retirar (*status quo*).

En la validación de la estructura interna, necesaria para verificar si los atributos de medición corresponden a los atributos teóricos^(21)^ y para el ajuste del modelo, se utilizaron pruebas múltiples, siguiendo las recomendaciones contemporáneas sobre evidencias de validez^(12)^. También se analizó si la estructura factorial estaba adecuadamente representada por su dimensionalidad^(21)^.

En la versión brasileña, el modelo de cuatro dimensiones no se reprodujo. La divergencia hallada en el análisis paralelo indicó la necesidad de pruebas adicionales de dimensionalidad, dado que la literatura recomienda no limitar este análisis a una sola técnica^(34)^. La unidimensionalidad se mantuvo y, en la AFE, el modelo unidimensional resultó ser el más adecuado, sin sustentar la permanencia de las dimensiones en la población de este estudio. Cabe resaltar que las pruebas aplicadas siguieron estándares robustos descritos en la psicometría contemporánea, consideradas de excelencia en el desarrollo y adaptación de instrumentos^(11-12)^.

En el análisis del instrumento original^(5)^ las autoras señalaron preliminarmente que la dimensión Capacidad de liderar probablemente no se mantendría en el modelo final, pues varios ítems parecían evaluar un aspecto limitado del constructo. La muestra del estudio original no alcanzó el número mínimo indicado en la literatura, con apenas 101 profesionales, aspecto que pudo influir en los resultados y explicar las diferencias entre ambos estudios. Otra diferencia es que no se realizaron análisis de dimensionalidad ni pruebas de AFE y AFC, optándose solo por el alfa de Cronbach, criterio considerado insuficiente en el presente estudio para evaluar la adecuación del modelo original.

En consecuencia, se aplicaron técnicas analíticas distintas de las del modelo original, reconociendo la existencia de métodos más modernos en la literatura especializada. A pesar de elegir procesos analíticos diferentes, se consideró la EALCI un instrumento fundamental, con aportes relevantes para la realidad y el contexto brasileño, dado su marco teórico, su amplia tradición y su aplicación en la práctica de constructos de interprofesionalidad^(2)^.

Es importante destacar que la versión japonesa^(27)^ tampoco reprodujo la misma dimensionalidad del AICLS original. Tras el análisis factorial exploratorio con los 28 ítems distribuidos en cuatro factores, el instrumento se redujo a diez ítems organizados en tres dimensiones, resultando en el AICLS-J aplicado a una muestra de 675 participantes.

Diferentes culturas pueden comprender, experimentar o expresar un mismo constructo de maneras distintas, lo que implica agrupar los ítems en dimensiones distintas a las observadas en la versión original. En la temática de la interprofesionalidad, dos instrumentos no reprodujeron la dimensionalidad original en el contexto brasileño. En la adaptación del *Readiness for Interprofessional Learning Scale* (RIPLS), la estructura original de cuatro factores no se replicó y el análisis factorial indicó una versión con 27 ítems distribuidos en tres dimensiones: trabajo en equipo y colaboración, identidad profesional y atención sanitaria centrada en el paciente^(35)^. En otro proceso de traducción y adaptación transcultural, la *Interprofessional Collaboration Measurement Scale* (IPC), los tres factores de la versión original no se replicaron en los análisis factoriales exploratorio y confirmatorio de la muestra brasileña, indicando un instrumento unidimensional^(36)^, resultado similar al obtenido en el presente estudio.

En ambos casos, la dificultad para reproducir las mismas dimensiones de la versión original puede atribuirse a las diferencias culturales, a los contextos educativos y a las prácticas distintas, dado que se trata de constructos con múltiples aspectos interdependientes. Bajo ese supuesto, esto puede ocurrir no solo entre países, sino también entre regiones de un mismo país y entre diferentes tipos de servicios de salud. Existe consenso en que las diferencias de dimensionalidad no deben considerarse una limitación, sino que pueden reflejar que, en el escenario de aplicación de la investigación, el constructo fue percibido como holístico, o incluso indicar lagunas en la formación y en la práctica interprofesional en salud^(36)^. Se refuerza, por tanto, y se corrobora las directrices del área, la necesidad de analizar la equivalencia de la estructura interna y de la medida de la escala considerando las diferencias regionales^(12)^, y que dicho análisis debe realizarse siempre que se opte por utilizar una escala de medición.

Se infiere así que el liderazgo colaborativo interprofesional puede verse influenciado por aspectos de la estructura organizacional, las relaciones de poder, la formación profesional y la cultura institucional, los cuales varían significativamente entre países, regiones y tipos de servicios. El proceso de adaptación permite que el instrumento sea sensible a las especificidades lingüísticas, culturales y sociales de su contexto, mantenga los fundamentos conceptuales y teóricos del instrumento original y contribuya al avance de la investigación y a la aplicabilidad de nuevos modelos de gestión basados en la organización interprofesional de los equipos.

En los análisis de evidencias de confiabilidad de la versión unidimensional brasileña de la EALCI, el coeficiente ômega de McDonald y el alfa de Cronbach fueron superiores a 0,70. La versión japonesa empleó solo el alfa de Cronbach, con valor = 0,987^(27)^. En el presente estudio se optó por presentar ambos indicadores para aumentar la precisión de la interpretación.

Al comparar el contexto de los ítems del instrumento CSLS^(8)^ con la versión brasileña de la EALCI, aunque no se evalúa la estructura jerárquica, se observa una proximidad entre los aportes teóricos de ambos instrumentos y entre los ítems, referentes a la colaboración entre los miembros del equipo con roles bien establecidos, fomentando la creatividad y las discusiones en la planificación de cuidados, así como el intercambio de conocimientos, habilidades y expertise según las necesidades de cada situación.

El instrumento traducido y validado al portugués corrobora la idea de que el liderazgo colaborativo es fundamental y prepara a los líderes de equipos interprofesionales para coordinar y colaborar, respetando al paciente y a la familia en sus propias decisiones de cuidado, como vía para ofrecer atención de salud de calidad y con seguridad. Trabajar de forma interprofesional y colaborativa requiere la corresponsabilización de todos por los resultados producidos^(5)^.

Contar en la literatura nacional e internacional con un instrumento capaz de medir adecuadamente el constructo de liderazgo colaborativo interprofesional representa un avance en el campo de la interprofesionalidad. Este instrumento puede utilizarse en programas de capacitación y formación profesional dirigidos a desarrollar dicha competencia, sobre todo desde la perspectiva del involucramiento de pacientes y cuidadores en los procesos de toma de decisiones, con vistas a mejorar la experiencia del paciente y la calidad y seguridad del cuidado, práctica que aún no se encuentra plenamente consolidada en los servicios de salud^(37)^.

Como limitaciones del estudio, se señala que existen pocos análisis del instrumento traducido a otros idiomas y que la propia versión canadiense contó con una muestra inferior a la recomendada en la literatura. Solo se identificó el estudio japonés^(27)^, y el presente trabajo intenta avanzar para suplir esa laguna, con futuras indicaciones de nuevas aplicaciones y análisis de la EALCI. Se desconocen otros instrumentos que midan el liderazgo colaborativo interprofesional o escalas similares validadas en Brasil, lo que impide comparaciones.

Aunque la dimensionalidad de la escala original no se haya replicado –aspecto que podría considerarse una limitación–, se enfatiza que se siguieron pruebas analíticas más contemporáneas y diferentes de las aplicadas originalmente, por considerarlas más pertinentes en este estudio para este tipo de escala. Entre las responsabilidades de los investigadores está presentar los resultados con transparencia y respetar los principios éticos que rigen la práctica científica, incluso cuando estos no coincidan con los de los estudios originales. Dichos resultados también contribuyen al avance del conocimiento y al perfeccionamiento de la escala que mide el liderazgo colaborativo interprofesional. Así, las limitaciones encontradas deben considerarse en futuras investigaciones y en la mejora de la EALCI.

Otra limitación es que no se analizaron las interacciones entre los profesionales que conformaban los equipos en las áreas de actuación del estudio, aspecto que podría configurar un análisis de validez de criterio. Por ello se señalan nuevas investigaciones, tanto de la aplicación del instrumento traducido y validado en otros contextos de práctica interprofesional como de la validez convergente^(38)^, para establecer la correlación del instrumento con herramientas que evalúan constructos similares, sobre todo con escalas de práctica colaborativa interprofesional ya validadas y ampliamente utilizadas.

Como implicaciones para la práctica, se recomienda que la EALCI sea utilizada en el contexto brasileño para la medición global del liderazgo colaborativo interprofesional y que se apliquen procedimientos de análisis contemporáneos en los diferentes contextos de atención de salud, con el fin de fortalecer su aplicabilidad.

## Conclusión

La EALCI, compuesta por 28 ítems, demostró ser un instrumento para medir el liderazgo colaborativo interprofesional, con estructura unidimensional y evidencias adecuadas de validez de contenido, proceso de respuesta, estructura interna y confiabilidad. Es una escala válida para realizar diagnósticos situacionales, apoyar políticas institucionales de intervención y evaluar el impacto de los programas educativos desde la perspectiva de la práctica interprofesional.

El liderazgo colaborativo es una competencia indispensable en el trabajo en equipo interprofesional, considerada esencial en la atención sanitaria para garantizar la centralidad del paciente y la familia. La disponibilidad de esta escala contribuirá a comprender los procesos implicados en esta forma de liderazgo, su presencia en los equipos interprofesionales y podrá ayudar a las instituciones de salud en los programas de desarrollo profesional, favoreciendo la implementación de este modelo asistencial de cuidado.

## Data Availability

Los conjuntos de datos relacionados con este artículo estarán disponibles previa solicitud al autor correspondiente.
